# A Pentatricopeptide Repeat Protein Restores Fertility in Tadukan‐Type Cytoplasmic Male Sterile Rice via the Cleavage of the Mitochondrial *orf312* RNA

**DOI:** 10.1111/ppl.70308

**Published:** 2025-05-29

**Authors:** Ayumu Takatsuka, Yuko Iwai, Hakim Mireau, Tomohiko Kazama, Hiroyuki Ichida, Tomoko Abe, Keisuke Igarashi, Kinya Toriyama

**Affiliations:** ^1^ Graduate School of Agricultural Science Tohoku University Sendai Miyagi Japan; ^2^ Institute Jean‐Pierre Bourgin for Plant Sciences (IJPB) Université Paris‐Saclay, INRAE, AgroParisTech Versailles France; ^3^ Graduate School of Agriculture Kyushu University Fukuoka Fukuoka Japan; ^4^ RIKEN Nishina Center for Accelerator‐Based Science Saitama Japan

**Keywords:** cytoplasmic male sterility, mitochondrial RNA processing, *Oryza sativa*, pentatricopeptide repeat protein, restorer of fertility

## Abstract

Cytoplasmic male sterility (CMS) is associated with the inhibition of pollen and/or anther development regulated by a CMS‐causing gene in the mitochondrial genome; it is a useful trait for preventing self‐pollination and producing F_1_ hybrids, which can boost crop yields. Pollen and/or anther development can be recovered by the action of the *RESTORER OF FERTILITY* (*Rf*) gene, a nuclear‐encoded gene. Most reported *Rf* genes encode pentatricopeptide repeat (PPR) proteins, which bind to RNA and promote RNA processing of the respective CMS‐causing gene. In this study, we report the map‐based cloning of the *Rf* gene (*Rfta*) for Tadukan‐type CMS (TA‐CMS) in rice ( *Oryza sativa* L.), with anther dehiscence and seed setting inhibited by the mitochondrial gene *orf312*. The *Rfta* locus was delimited to a region comprising 10 PPR genes forming a cluster on chromosome 10. The complementation test revealed that the introduction of a PPR gene, *PPR796*, into the TA‐CMS line resulted in the recovery of the anther dehiscence and seed setting. RNA‐gel blot analysis and the determination of 3′ ends of the *orf312* RNA confirmed the *PPR796*‐mediated cleavage of the *orf312* RNA in the transgenic TA‐CMS line. Furthermore, RNA gel electrophoretic mobility shift assays revealed that the recombinant PPR796 protein bound to the 3′ side of the *orf312* RNA in vitro. We concluded that RFta/PPR796 binds to *orf312* RNA and promotes RNA cleavage to restore fertility in TA‐CMS.

## 1 Introduction

Cytoplasmic male sterility (CMS) is effective for commercial production of F_1_ hybrid seeds and prevention of self‐fertilization. In most CMS plants, the mitochondrial genes inhibit pollen and/or anther development. After the discovery of T‐type CMS and associated genes in maize (Rhoades 1931; Warmke and Lee 1978), various CMS plants and mitochondrial CMS‐causing genes have been identified (Hanson and Bentolila 2004; Chen and Liu 2014, for reviews).

A CMS trait is recovered by nuclear genes called *RESTORER OF FERTILITY* (*Rf*) (Schnable 1998). Most *Rf* genes encode pentatricopeptide repeat (PPR) proteins, which are involved in RNA processing such as RNA editing, splicing, RNA stabilization, maturation and translation (Kazama and Toriyama 2003; Schmitzlinneweber and Small 2008; Gaborieau et al. 2016; Wang et al. 2021). Fertility is mostly restored in CMS lines by suppressing the CMS‐causing gene expression by the RF protein. PPR proteins consist of repeated motifs, each comprising 35 amino acids, which mediate specific association with individual RNA bases. The RNA nucleotide recognized by each PPR motif can be predicted based on the combination of amino acid residues present at the 5th and 35th positions within each repeat (Barkan et al. 2012; Cheng et al. 2016). These positions correspond to 4th and ‘ii’ amino acids in the Yagi et al. (2013) nomenclature.

To date, molecular cloning of mitochondrial CMS‐causing genes and nuclear *Rf* genes has been carried out in various species, including rice ( *Oryza sativa* L., see Toriyama 2021, for a review). For instance, a mitochondrial gene *orf79* and nuclear genes *Rf1a* and *Rf1b* in Chinsurah‐Boro/Taichung 65 (T65)‐type CMS (BT‐CMS, Iwabuchi et al. 1993; Kazama et al. 2008; Wang et al. 2006), *orfH79*, *Rf5*, and *Rf6* in Hong Lian‐type CMS (HL‐CMS, Peng et al. 2010; Hu et al. 2012; Huang et al. 2015), L‐*orf79* and *Rf2* in Lead Rice‐type CMS (LD‐CMS, Itabashi et al. 2009; Itabashi et al. 2011), *WA352*, *Rf3*, and *Rf4* in Wild Abortive‐type CMS (WA‐CMS, Balaji Suresh et al. 2012; Luo et al. 2013; Kazama and Toriyama 2014) have been reported.

WA‐CMS is the most widely used CMS system for F_1_ hybrid rice production (Li et al. 2007; Huang et al. 2014). Pollen abortion occurs at the uninucleate microspore stage, and by the flowering stage, pollen grains are irregularly shaped and lack starch accumulation. The mitochondrial CMS gene *WA352* is a chimeric gene composed of *orf284*, *orf224*, *orf288*, and an origin‐unknown sequence, and it is co‐expressed with the upstream gene encoding ribosomal protein large subunit 5 (*rpl5*, Luo et al. 2013). The WA352 protein in WA‐CMS has been reported to carry a C‐terminal domain that interacts with the cytochrome c oxidase subunit 11 (COX11) and to induce a burst of reactive oxygen species, resulting in premature tapetal programmed cell death and, consequently, sporophytic male sterility (Luo et al. 2013). Furthermore, CMS‐causing genes or candidate genes that showed high similarity to the COX11‐interacting region of WA352 have also been reported in other rice CMS types: *orf352* in RT102‐type CMS (Okazaki et al. 2013), *orf312* in Tadukan‐type CMS (Takatsuka et al. 2021) and *orf288* in T65/ *O. glaberrima* ‐type CMS (Toriyama et al. 2024).

Previous reports reflect the fertility restoration in WA‐CMS controlled in a sporophytic manner. *Rf3* and *Rf4* genes for WA‐CMS have been mapped to chromosomes 1 and 10, respectively (Balaji Suresh et al. 2012). *Rf4* has been shown to function post‐transcriptionally and promote RNA degradation of the *WA352* transcripts. RF3 protein has been shown to function post‐translationally (Luo et al. 2013). The *Rf4* gene for WA‐CMS has been cloned; it encodes a 782‐aminoamino‐acid protein containing 18 repeats of the PPR motif (Kazama and Toriyama 2014; Tang et al. 2014). The molecular cloning of *Rf3* has not yet been reported.

In this study, we focused on the Tadukan‐type CMS (TA‐CMS, Takatsuka et al. 2021). In TA‐CMS, the pollen develops normally, but the anthers do not dehisce, leading to little or no seed set. A loss‐of‐function analysis of *orf312*, a candidate CMS‐associated gene in the Tadukan mitochondrial genome, using Mito‐TALEN technology demonstrated that *orf312* is responsible for the anther indehiscence in TA‐CMS (Takatsuka et al. 2022).

A rice CMS line carrying the *orf312* gene has been reported in Tetep‐CMS, which exhibited an abnormal anther dehiscence phenotype, lacking the ability to release pollen grains as in TA‐CMS (Jin et al. 2021; Lee et al. 2022). Histological analysis of Tetep‐CMS revealed that the cavity formation at the breakage site, which is crucial for anther dehiscence, was arrested during the early stage of anther development. The *Rf* gene for Tetep‐CMS, named *Rf‐Tetep*, has been mapped to a region on chromosome 10, harboring several restorer genes, *Rf1a*, *Rf1b*, and *Rf4* (Jin et al. 2021). The *Rf‐Tetep* candidate gene was suggested to be *ORF6/PPR1* encoding a PPR domain‐containing protein (Lee et al. 2022). However, this candidate gene has not yet been validated as *Rf‐Tetep* based on complementation tests.

Here, we carried out map‐based cloning of an *Rf* gene for TA‐CMS, named ‘*Rfta*’, derived from the Tadukan genome. Complementation tests demonstrated that *Rfta* was *PPR796*, encoding a PPR protein of 796 amino acids. *PPR796* has been shown to promote the cleavage of th*e orf312* RNA and contributes to the anther dehiscence and spikelet fertility.

## 2 Materials and Methods

### 2.1 Plant Materials and Culture Conditions

The CMS line (TAA), restorer line (TAR), maintainer line (Taichung 65: T65), and Tadukan were propagated and grown following a previously described method (Takatsuka et al. 2021). Transgenic plants were grown in a greenhouse under temperature‐ and day/night‐regulated conditions (Takatsuka et al. 2022). Seed setting rates were expressed as the average values recorded using three panicles of each plant.

### 2.2 Mapping of *Rfta*

The genotypes of the SSR and InDel markers were determined using the primers listed in Table S1. DNA was extracted from leaves, followed by PCR analysis, and polyacrylamide gel electrophoresis was performed as described previously (Kazama and Toriyama 2003). For whole genome sequencing (WGS) of the Tadukan genome, high‐quality DNA was extracted from young leaves using the NucleoBond High Molecular Weight DNA Kit (Macherey‐Nagel). HiFi reads > 10 kb from the PacBio Sequel II sequencer were assembled under 1 kb‐overlapped conditions. Gene structures and functions in this sequence were predicted using MEGANTE (Numa and Itoh 2014; https://megante.dna.affrc.go.jp/) or homology research using the sequences of *Rf*‐like PPR genes as queries. The structure and number of the PPR motif in each PPR protein were predicted using PPRFinder (Cheng et al. 2016; Gutmann et al. 2020; https://ppr.plantenergy.uwa.edu.au/).

### 2.3 Expression Analysis

RNA was extracted from spikelets containing anthers at the meiotic stage and from anthers at the flowering stage using the RNeasy Plant Mini Kit (QIAGEN). RNA‐gel blot analysis was performed following a previously described method (Takatsuka et al. 2021) with some modifications. The gel recipe was modified to include 2% agarose and 2% formaldehyde for RNA gel electrophoresis. The RNA‐transferred membranes were hybridized with the *orf288* DNA probe (Takatsuka et al. 2021) at 63°C in a buffer containing DIG Easy Hyb powder. The band size was determined by plotting the measured distance against the known sizes of the marker bands on a logarithmic scale. RT‐PCR analysis was performed using the PrimeScript RT‐PCR Kit (TaKaRa Bio.). Oligo primers were designed to specifically match the target PPR gene while introducing mismatches with other homologous PPR genes, ensuring selective binding to the target PPR gene (Hayashi et al. 2004).

### 2.4 Vector Construction and Transformation

The genomic fragments of PPR genes, including 1.7–2.0 kb of upstream regions and 0.2–1.8 kb of downstream regions, were amplified using PrimeSTAR GXL SP DNA Polymerase (TaKaRa Bio.) and cloned into a pCR‐Blunt vector. The CDS sequences inserted into the vectors were validated through sequencing analysis. The PPR genes were then subcloned into the binary vector pZH2B (Kuroda et al. 2010) at the *Sal*I or *Bam*HI sites by incorporating these restriction sites into the oligo primers in advance (Table S2). The amplicons of the *PPR796*, *PPR782*, and *PPR794* fragments were obtained by 2nd nested‐PCR using the 1st‐PCR products as the template DNA. The resulting binary vectors were introduced into TAA through the *Agrobacterium*‐mediated transformation (Takatsuka et al. 2022).

### 2.5 Determination of the 3′ Ends of *orf312* RNA

Rapid amplification of cDNA ends (RACE) analysis was conducted to detect the position of the 3′ terminal site of the *orf312* transcript. RNA was extracted from the spikelets of TAA, TAR, and a *PPR796*‐transgenic line, which contained anthers at the meiotic stage, followed by DNase treatment; 3′ RACE was performed following the instructions provided along with the SMARTer RACE 5′/3′ Kit (TaKaRa Bio.) after treating the RNA with *Escherichia coli* Poly(A) Polymerase (New England Biolabs). The 3′ RACE‐PCR primers specific to the *orf312* sequence were extended to harbor the sequence for In‐Fusion HD Cloning (TaKaRa Bio.) (Table S2); after cloning the amplicon into the pRACE vector, the vector was introduced into Stellar Competent cells (TaKaRa Bio). At least 15 clones were sequenced for each line.

### 2.6 Prediction of PPR‐Binding Sites

A mitochondrial targeting signal peptide was predicted by MitoFates (Fukasawa et al. 2015; https://mitf.cbrc.pj.aist.go.jp/MitoFates/cgi‐bin/top.cgi) and removed from the amino acid sequences of a PPR protein before prediction of PPR motifs. The truncated sequence was uploaded to the PPRFinder server (Cheng et al. 2016; Gutmann et al. 2020; https://ppr.plantenergy.uwa.edu.au/) to extract each PPR code. The RNA base preferences of PPR796 were estimated following the 2‐letter code and the probability score established by Kobayashi et al. (2019) and depicted using the Logomaker package (https://logomaker.readthedocs.io/en/latest/). PPR‐binding sites were predicted within a sequence ranging from 72 bp upstream to 33 bp downstream of the CDS (from −72 to +972 counting from the initiation codon) of *orf312* using FIMO MEME Suite 5.5.5 (https://meme‐suite.org/meme/tools/fimo). Among the predicted PPR796‐binding sites, we extracted those with the + direction strand (sequence in the same direction as the mRNA sequence) and a *q*‐value < 0.999.

### 2.7 RNA Electrophoresis Gel Mobility Shift Assay (REMSA)

The *PPR796* CDS, lacking the N‐terminal signal sequence, was amplified by PCR and cloned into pDONR207 via the Gateway (Invitrogen) BP reaction (Table S2). The resulting *PPR796* sequence was subcloned by an LR reaction into pDEST17 (Invitrogen) to generate a translational fusion with a 6 x Histidine‐tag sequence. The resulting recombinant PPR protein was expressed in the *E. coli* BL21 (DE3) strain (New England Biolabs), extracted, and purified for gel shift assay as previously described (Wang et al. 2021). DNA fragments in or near the *orf312* CDS region, with sizes of less than 200 bp, were amplified by PCR using primers that included the T7 promoter sequence (Table S2). The resulting DNA fragments were in vitro transcribed using T7 RNA Polymerase (Roche) and the DIG RNA Labeling Mix (Roche) to label the generated probes. The generated RNA probes were gel‐purified and used for gel shift assays as described in Wang et al. (2021). Ribonucleotide Solution Mix (New England Biolabs) was used for the non‐labeled competitor probe.

## 3 Results

### 3.1 Fine‐Mapping of the *Rfta* Candidate Region Derived From Tadukan

The Tadukan‐type CMS line (TAA) and near‐isogenic restorer line (TAR) were derived from the BC_11_F_1_ and BC_3_F_3_ generations, respectively, of Tadukan (a cytoplasmic donor) backcrossed with Taichung 65 (T65; a recurrent pollen parent) (Takatsuka et al. 2021). TAR was selected based on the Tadukan homozygous genotypes of the SSR markers, SSR H10023 and SSR H10045, which flanked the *rf1a* locus on chromosome 10 (Takatsuka et al. 2021). TAA exhibited an indehiscent anther phenotype, resulting in no or very few seeds, with seed setting rates of 0 to 3%, while TAR showed a dehiscent anther phenotype with seed setting rates of approximately 90% (Table 1, Table S3), as previously reported in Takatsuka et al. (2021).

**TABLE 1:** Chromosomal position of markers in Nipponbare genome (IRGSP‐1.0), and genotypes and seed‐setting rates in TAA, TAR, and recombinant plants.

Position (kb)	Marker	TAA	TAR	BC_3_F_4_	BC_3_F_5_	BC_3_F_5_	BC_3_F_4_	BC_3_F_5_	BC_3_F_5_	BC_3_F_4_
				66_18	66_18_16	66_18_40	66_36	66_36_2	66_36_4	66_5
Seed setting rates (%)		1.4	93.7	87.6	79.1	72.1	3.2	0.4	0.0	0.0
18,006	SSR 1041	A	A	A	A	A	A	A	A	A
18,572	SSR 10081	A	A	H	H	A	A	A	A	H
18,727	SSR 10085	A	T	H	H	H	A	A	A	A
18,746	SSR H10004	A	T	H	H	H	A	A	A	A
18,883	SSR H10023	A	T	H	H	H	A	A	A	A
18,976	SSR H10040	A	T	H	H	H	A	A	A	A
19,006	SSR H10045	A	T	H	H	H	A	A	A	A
19,083	SSR H10070	A	T	H	H	H	A	A	A	A
19,120	TTRf‐indel19,120	A	T	H	H	H	A	A	A	A
19,134	TTRf‐indel19,134	A	T	H	H	H	H	T	T	A
19,533	KNJI‐indel759	A	T	H	H	H	H	T	T	A
20,033	SSR 1061	A	T	T	T	T	T	T	T	T
20,347	SSR 1062	A	T	T	T	T	T	T	T	T
21,863	SSR 1065	A	T	T	T	T	T	T	T	T
23,115	SSR 1069	A	T	T	T	T	T	T	T	T

*Note:* A, H, and T indicate T65‐homozygous, T65 and Tadukan‐heterozygous, and Tadukan‐homozygous genotypes, respectively.

We used the siblings of TAR as the starting point of the mapping population; we investigated the genotypes of SSR and In/Del markers and their correlation with the anther phenotype and seed setting rates. As expected from the indehiscent/dehiscent anther phenotypes, the BC_3_F_4_ and BC_3_F_5_ generations showed a sporophytic mode of segregation between fertile and sterile plants. For fertile recombinant plants (Nos. 66_18, 66_18_16, and 66_18_40 in Table 1, Table S3), a region corresponding to homozygous or heterozygous Tadukan genotypes was considered to be essential for fertility restoration. For male sterile recombinant plants (Nos. 66_36, 66_36_2, 66_36_4, and 66_5 in Table 1, Table S3), exhibiting indehiscent anther phenotypes and no or very few seeds, a region carrying the homozygous T65 genotype was identified as the *Rfta* candidate region. Taken together, the *Rfta* candidate region was narrowed down to a region flanked by the DNA markers, SSR 10081 and TTRf‐indel 19,134 (Table 1). These map positions corresponded to 18,572 kb and 19,134 kb positions in the Os‐Nipponbare‐Reference‐IRGSP‐1.0 genome (Kawahara et al. 2013; Sakai et al. 2013).

The effect of the *Rfta* candidate region on *orf312* RNA processing was examined via RNA‐gel blot analysis using RNA extracted from anthers at the flowering stage (Figure S1). A single strong band corresponding to the *orf312* RNA was observed at 1.15 kb in sterile recombinant plants (Nos. 66_36_2 and 66_36_4) exhibiting the T65 homozygous genotype for the candidate region, as detected in TAA plants, while the band intensity was greatly reduced in TAR and the fertile plants (Nos. 66_18_16 and 66_18_40) possessing the Tadukan heterozygous genotypes for the candidate region. These results confirmed that the *Rf* gene regulating the expression of *orf312* was located in the delimited *Rfta* candidate region.

### 3.2 PPR Genes as Candidates for *Rfta*

**FIGURE 1:**
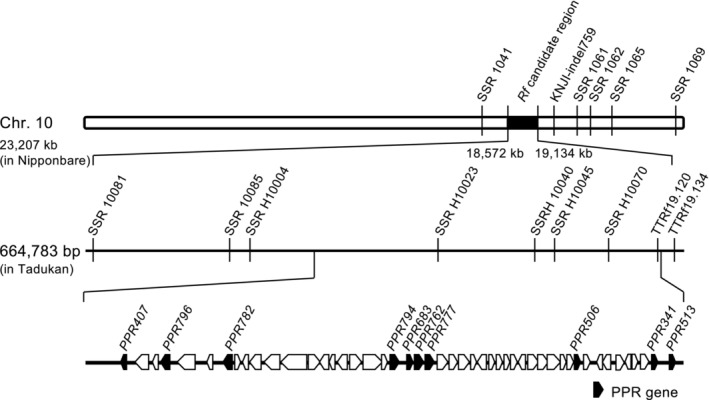
Schematic structure of chromosome 10 and fine mapping of the *Rfta* gene. The DNA molecular markers are listed in Table 1. The chromosomal positions are indicated based on the Nipponbare genome (IRGSP‐1.0). The black segment indicates the *Rfta* candidate region. Each box indicates a predicted gene and its orientation. Black boxes represent the PPR genes listed in Table 2.

We explored the *Rfta* candidate genes in the Tadukan genome after determining the whole genome sequence (WGS) using PacBio HiFi reads. The *Rfta* candidate region flanked by SSR10081 and TTRf‐indel19,134 was 664,783 bp in length, and 81 genes were predicted using MEGANTE, a web service tool for integrated plant genome annotation (Figure 1, Table S4).

We identified several PPR genes forming a gene cluster in this region; this *PPR* gene cluster was also present in the corresponding regions on chromosome 10 of other CMS restorer lines of rice, from which some PPR genes were cloned as *Rf* genes, such as *Rf1a* and *Rf1b* for BT‐CMS, *Rf5* for HL‐CMS, *Rf4* for WA‐CMS, and *Rf*_*98*_ for RT98‐CMS (Toriyama 2021, for a review). Moreover, RNA‐gel blot analysis of the Tadukan × T65 recombinant lines validated the involvement of the candidate gene in RNA processing of the mitochondrial gene *orf312* (Figure S1). Hence, we considered PPR genes in this cluster promising candidates for *Rfta* genes and focused on them.

Within the candidate region, seven *orf*s—named *PPR794*, *PPR683*, *PPR762*, *PPR777*, *PPR506*, *PPR341*, and *PPR513—*were predicted to encode PPR proteins using MEGANTE (Table S4). Additionally, three other PPR genes—*PPR407*, *PPR796*, and *PPR782*—were predicted through a homology search against *Rf*‐like PPR gene sequences. The subcellular localization of each PPR protein was predicted using WoLf PSORT (https://wolfpsort.hgc.jp), which suggested that all PPR proteins except for PPR341 could be localized to the mitochondria (Table 2). PPRFinder (https://ppr.plantenergy.uwa.edu.au/) predicted that all of these were P‐type PPR proteins. PPR proteins bind RNA on the rule that one PPR motif recognizes one RNA base residue (Schmitzlinneweber and Small 2008).

**TABLE 2:** PPR genes predicted in the *Rfta* candidate region.

*PPR*s	Position (kb)^a^	No. of PPR motif^b^	Subcellular localization (predicted by Wolf PSORT)	Identitiy with other PPR (a.a.)	
				Other PPR (feature)	Ideitical pairs/Length
					(Identity %)
*PPR407*	271,135—269,912	9	mito: 5.5, chlo_mito: 5.5, chlo: 4.5, cyto: 3.0	ZH11_PPR10‐454‐Z (Rf4‐like)	393/402 (97)
*PPR796*	299,545—297,155	19	mito: 9.5, cyto_mito: 5.5, chlo: 4.0	MH63_PPR9‐782‐M (Rf4)	756/769 (98)
*PPR782*	340,916—338,568	19	chlo: 8.5, chlo_mito: 6.8, mito: 4.0, cyto_mito: 2.8	MH63_PPR9‐782‐M (Rf4)	746/782 (95)
*PPR794*	449,683—452,067	18	mito: 9.5, cyto_mito: 5.5, chlo: 4.0	Np_Os10g0497300 (rf1a)	792/794 (99)
*PPR683*	460,462—462,513	16	chlo: 9.5, chlo_mito: 7.3, mito: 4, cyto_mito: 2.8	IR24_PPR791 (Rf1a, Rf5)	590/637 (92)
*PPR762*	464,833—467,121	19	mito: 9, chlo_mito: 7.3, cyto_mito: 5.3, chlo: 4.5	Np_Os10g0497300 (rf1a)	697/761 (91)
*PPR777*	472,831—475,164	19	mito: 7.5, chlo: 6, cyto_mito: 4.5	MH63_PPR9‐782‐M (Rf4)	722/776 (93)
*PPR506*	572,162—573,682	12	mito: 8.5, chlo_mito: 7.5, chlo: 5.5	IR24_PPR506 (Rf1b)	506/506 (100)
*PPR341*	624,668—625,693	7	cyto: 10, nucl: 2, chlo: 1	Np_PPR‐containing, At2g20710	337/341 (99)
*PPR513*	632,483—632,791; 632,876—634,108	9	chlo: 7.5, chlo_mito: 7.5, mito: 6.5	Np_PPR‐containing, At2g20710	507/513 (99)

^a^ The position in the 664,783‐kb *Rfta* candidate region (DDBJ accession No. LC868044).

^b^ Number of PPR motifs was predicted by PPRFinder.

Among these PPR genes, we explored strong candidates for *Rfta*. We set the criteria that the number of PPR motifs should be 10 or more, as suggested in a study of *Rf‐*like PPR genes (Melonek et al. 2016). Based on the number of PPR motifs predicted by the PPRFinder (Table 2), we selected the *PPR796, PPR782, PPR794, PPR683, PPR762, PPR777*, and *PPR506* proteins. Then, *PPR506* was excluded from the candidates because its amino acid sequence was completely identical to that of PPR506/RF1b, which was known to function in the degradation of the *orf79*‐containing RNA, the transcript responsible for the BT‐CMS (Wang et al. 2006). The proteins encoded by the selected PPR genes, *PPR796, PPR782, PPR794, PPR683, PPR762*, and *PPR777*, show high identities to known *Rf*‐like PPR proteins (Table 2). In particular, PPR796 exhibited 98% identity in its amino acid sequence to that of RF4 for WA‐CMS (Table 2, Figure S2). Because RF4 has been reported to promote RNA degradation of *WA352*, the CMS‐causing gene of WA‐CMS, which carries the 3′ half of the CDS encoding the COX11‐interacting region, is almost identical to that of *orf312* (Takatsuka et al. 2021). *PPR796* was considered the strongest candidate for the *Rfta* gene that promotes RNA processing of *orf312*.

**FIGURE 2:**
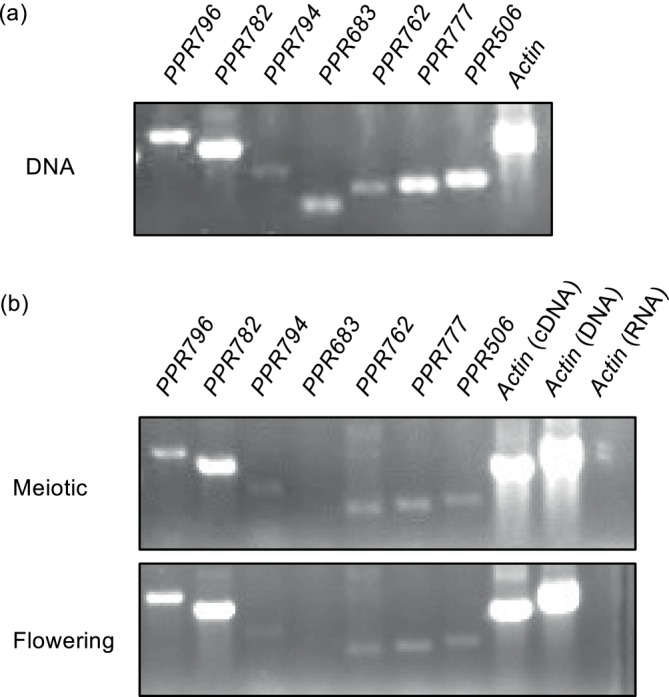
The expression of PPR genes in anthers of TAR. (a) Genomic PCR to confirm amplification of the sequence specific to each PPR gene. (b) RT‐PCR. cDNA was synthesized using oligo d(T) primers and RNA isolated from spikelets containing anthers at the meiotic stage and anthers at the flowering stage; the *Actin* sequence was considered the control to confirm the absence of genomic DNA contamination in RNA and cDNA.

As the *Rfta* gene was expected to be expressed in anthers at the meiotic and/or flowering stages, we examined the expression of the six *Rfta* candidates using RT‐PCR. Since the sequences of those PPR genes are highly similar, we designed specific primers to amplify each PPR gene separately and confirmed the specific amplification of each gene by sequencing the PCR products obtained from genomic DNA. RT‐PCR demonstrated that, except for *PPR683*, the five PPR genes (*PPR796, PPR782, PPR794, PPR762*, and *PPR777*) were expressed in both the meiotic and flowering stages (Figure 2), thus indicating that these five PPR genes were good *Rfta* candidates.

### 3.3 Introduction of *PPR796* Recovered the Fertility of TA‐CMS

**FIGURE 3:**
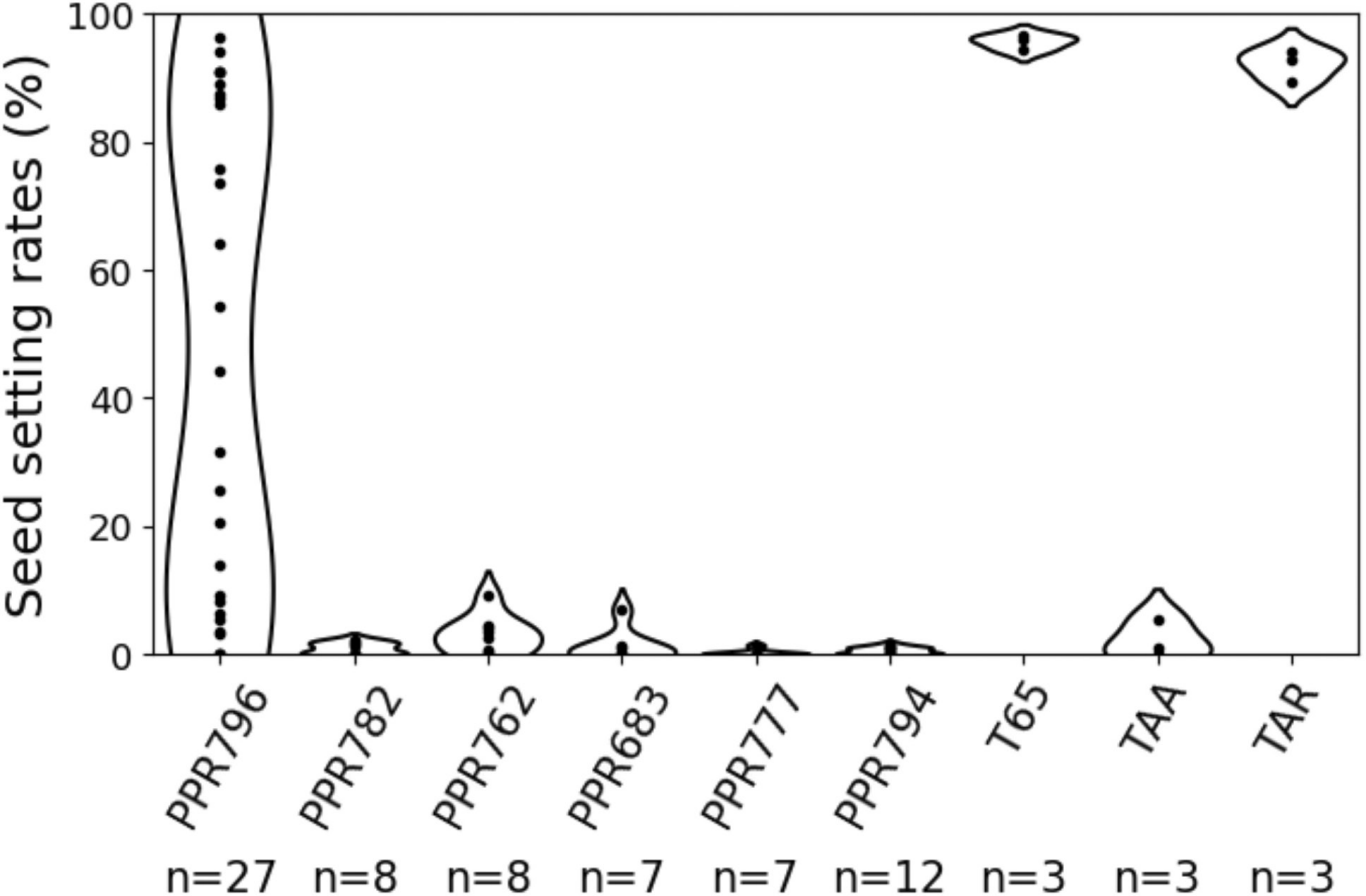
Scatter and violin plot for seed setting rates of all transgenic TAA plants with each PPR gene in the complementation test. Every individual plant is derived from an independently transformed callus. *n* = the number of plants observed in each line (the anther phenotypes and seed setting rates for individual plants are shown in Figures S4–S6 in Table S5.)

Genomic fragments of the five PPR genes, *PPR796*, *PPR782*, *PPR794*, *PPR762*, and *PPR777* (Figure S2) were introduced into TAA to identify the PPR gene responsible for anther dehiscence and the restoration of spikelet fertility. The genomic fragment of *PPR683* was considered a negative control, as it was not expected to promote fertility restoration in TAA plants due to the absence of *PPR683* expression in anthers (Figure 2). Anther dehiscence and fertility restoration were observed in the transgenic *PPR796‐*expressing TAA (*PPR796*‐transgenic) plants (Figure 3). The seed setting rates of panicles in *PPR796*‐transgenic plants ranged from 0% to 96%, with four plants (#1, #7, #8, and #21) setting over 90% of seeds, similarly to the restorer line TAR (Figure 3, Table S5). Out of 27 independent *PPR796*‐transgenic plants, 21 exhibited anther dehiscence, while seven plants (#3, #6, #9, #13, #14, #18, and #26) retained an anther indehiscence phenotype with seed setting rates of less than 10% (Figure 3, Figure S4, Table S5). Contrastingly, the other four PPR genes (*PPR782*, *PPR794*, *PPR762*, and *PPR777*) did not revert the indehiscence phenotype; eight *PPR782*‐transgenic plants, eight *PPR762*‐transgenic plants, and seven *PPR777*‐transgenic plants retained indehiscent anthers, with seed setting rates of less than 10%, similar to TAA (Figure 3, Figures S5 and S6, Table S5). Seed setting restoration was also not observed in *PPR683*‐transgenic plants (Figure 3, Figure S5, Table S5). We, therefore, concluded that *PPR796* is the *Rfta* gene responsible for fertility restoration in TA‐CMS.

**FIGURE 4:**
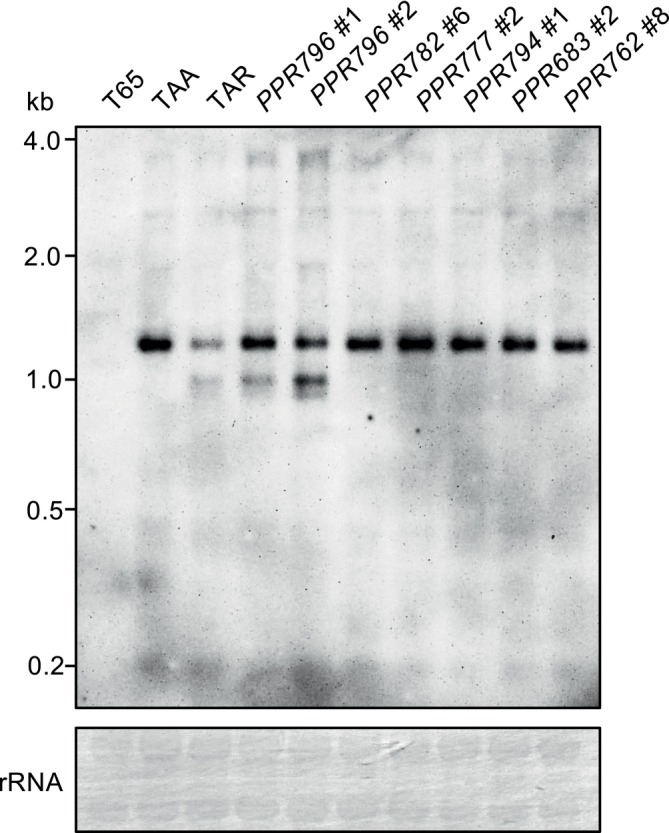
Expression profile of *orf312* in *PPR*‐transgenic TAA lines compared with that in TAA and TAR. RNA‐gel blot analysis of representative *PPR*‐transgenic plants. Methylene blue‐stained rRNA was used as the loading control.

Next, we investigated whether the *Rfta/PPR796* was responsible for *orf312* RNA processing through RNA‐gel blot analysis, in which the gel recipe and hybridization conditions were slightly modified to achieve sharper hybridization signals (see Materials and Methods). The *orf312* expression patterns in the sterile transgenic plants, *PPR782* #6, *PPR777* #2, *PPR794* #1, *PPR683* #2, and *PPR762* #8, were the same as those recorded in TAA (Figure 4). However, fertile transgenic plants, *PPR796* # 1 and *PPR796 # 2*, showed additional bands at 0.95 kb with 1.15‐kb as seen in TAR plants (Figure 4). These results reflect the involvement of *PPR796* in *orf312* RNA processing, implying cleavage of the 1.15‐kb *orf312* RNA into a truncated 0.95‐kb RNA.

Noticeably, 1.15 kb‐*orf312* RNA in the *PPR796*‐transgenic lines was not so drastically decreased as observed in TAR (Figure 4), suggesting the presence of an additional *Rf* factor other than *PPR796* in TAR to degrade the *orf312* RNA.

### 3.4 PPR796 Protein Binds 3′ Terminal Side of *orf312* RNA In Vitro

**FIGURE 5:**
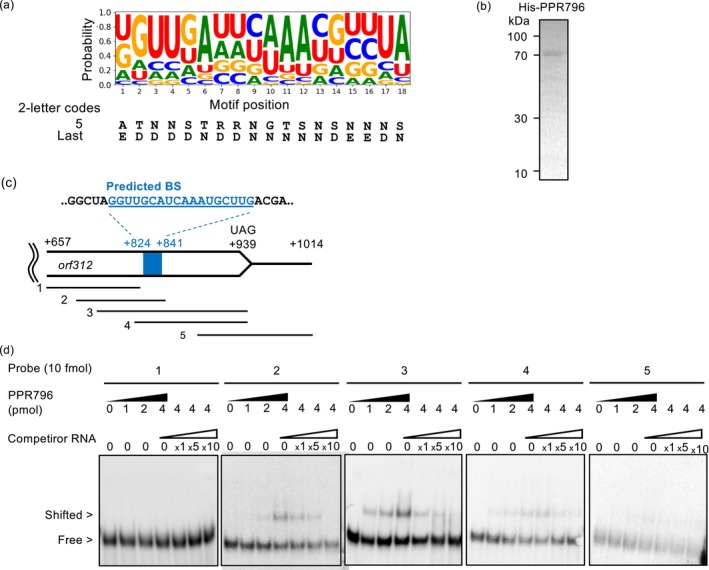
Binding test of PPR796 protein and *orf312* RNA. (a) Base preference of PPR796 predicted by amino acid residues for 2‐letter PPR code in each PPR motif. (b) Coomassie brilliant blue staining of purified histidine tag‐recombinant PPR796 protein. (c) The predicted binding site (BS) at 3′ sides of the *orf312* transcript. The position of probes used for RNA gel electrophoretic mobility shift assays (REMSA) is indicated using parallel lines. (d) REMSA of PPR796 and *orf312* RNA. The bound RNA was shifted up from the band of the free probe. Competitor RNA represents a non‐labeled RNA probe. PPR796 protein and competitor RNA were loaded into each lane in different dosages.

Most PPR proteins bind to RNA molecules in a sequence‐specific manner (Schmitzlinneweber and Small 2008). The sequences recognized by pentatrico/long/short (PLS)‐class PPR in the mitochondria and chloroplasts were deeply analyzed in *Arabidopsis thaliana* , which was applied to the establishment of a computational system to predict the binding base preference of PPR repeats (Kobayashi et al. 2019). We used the established PPR recognition code to predict the binding preference of PPR796, which was predicted to carry 18 PPR motifs after removing the mitochondrial targeting signal sequence (Figure 5a, Figure S2). Within the *orf312* transcript (from −72 to +972) reported by Jin et al. (2021), the promising binding sequences (BSs) were predicted mainly at the 3′ terminal side of the *orf312* CDS in the transcript (Table S6).

To confirm the binding between PPR796 and the predicted BS of the *orf312* RNA, we performed RNA gel electrophoresis mobility shift assay (REMSA). The PPR796 protein with a 6 x histidine tag was expressed in *E. coli* and purified for REMSA (Figure 5b). The 18 nt sequence, spanning nucleotides +824 to +841 on the 3′ side of orf312 and exhibiting the lowest *p*‐value among all predicted binding sites, was tested for interaction with PPR796 (Figure 5c, Table S6). Shifted bands were observed for probes 2, 3, and 4, which contained the predicted BS, but not for probes 1 and 5, which lacked that sequence (Figure 5d). The signal of shifted bands increased with the added amount of PPR796 protein and, in contrast, decreased as the amounts of non‐labeled competitor RNAs increased. These results suggest PPR796 may bind to the 3′‐region of the *orf312* CDS.

### 3.5 The *orf312* Transcript Is Truncated Within Its CDS in TAR and *PPR796* ‐Transgenic Lines

**FIGURE 6:**
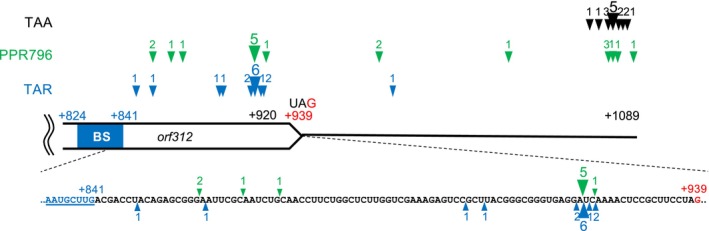
The terminal sites of *orf312* transcript detected by 3′ RACE in TAA, TAR and *PPR796*‐transgenic plant. The position on the *orf312* transcript was determined relative to the initiation codon of *orf312*. Arrowheads and numbers indicate the detected terminal sites and number of clones obtained by Rapid amplification of cDNA ends (RACE) analysis (see Table S7). Black, green and blue arrowheads and numbers correspond to TAA, *PPR796*‐transgenic plants and TAR, respectively. The sequence around the main terminal sites is shown in a schematic diagram. PPR796‐binding site (BS) is shown in Figure 5.

As indicated by RNA‐gel blot and REMSA analyses (Figures 4 and 5), PPR796 appears to cleave the *orf312* RNA within the 3′ region of its CDS. The positions of the 3′ terminal ends of the *orf312* mRNA were determined by rapid amplification of cDNA ends (RACE) in TAA, TAR, and the transgenic *PPR796*#2 plant. The 3′ terminal end of *orf312* was primarily detected at the +1089 nt position in the TAA plant, whereas it was most frequently located at the +920 nt position in TAR and the *PPR796*‐transgenic plants (Figure 6, Table S7). These results strongly suggest that PPR796 induces cleavage at the +920 nt position within the *orf312* transcript.

According to the RACE analysis, the cleavage at the +920 nt position would produce a truncated RNA measuring 0.958 kb. This estimate aligns with the truncated RNA produced by PPR796 and detected by RNA‐gel blot analysis (Figure 4). The convergence of these two experimental results strongly supports that PPR796 induces cleavage within the *orf312* RNA at the +920 nt position.

## 4 Discussion

TA‐CMS in this study and Tetep‐CMS analyzed by Lee et al. (2022) were expected to have the same *Rf* gene because both possess the identical *orf312* gene (Takatsuka et al. 2022). We identified the *PPR796* gene as the *Rf* gene for TA‐CMS. The same *PPR796* gene has been identified in the Tetep genome approximately 50 kb away from the delimited 109‐kb candidate region for *Rf‐Tetep*. *ORF6/PPR1*, at the locus corresponding to *Os10g0493100* in the Nipponbare genome (IRGSP‐1.0), was reported as the candidate gene for *Rf‐Tetep*. Our mapping analysis included the *ORF6/PPR1* gene as Gene_21.1 (Table S4); however, we did not test the Tadukan allele of this gene because it was not selected as a PPR gene in our study, which was potentially attributable to only two PPR motifs in it. The *ORF6/PPR1* gene may serve as a co‐factor of fertility restoration in TA‐CMS, as RNA‐gel blot analysis (Figure 4) revealed that the signal intensity of the 1.15‐kb band of the *orf312* RNA in the *PPR796*‐transgenic plants (PPR796#1 and PPR796#2) was not as considerably decreased as that in TAR, suggesting that an additional gene other than *PPR796* might have a role in reinforcing the degradation of the *orf312* RNA.

RNA‐gel blot analysis, REMSA analysis, and determination of the 3′ ends of the *orf312* RNA suggest that RFta/PPR796 binds in the 3′ region in the *orf312* RNA and possesses an ability to induce partial cleavage near this region in the *orf312* RNA (Figures 4, 5, 6). The ability of PPR796 to induce cleavage was also supported by the fact that it contains a Restorer‐of‐fertility C‐terminal domain (RfCTD), which is correlated with the ability of Rf‐like proteins to mediate cleavage in their mitochondrial RNA targets (Huynh et al. 2023). As shown in Figure S2, the C‐terminal sequence of RFta/PPR796 is highly similar to the sequence of RfCTD of RF1a, which is known to promote the cleavage of the *orf79*‐containing RNA in BT‐CMS (Kazama et al. 2008; Wang et al. 2006). The RfCTD of RFta/PPR796 differs significantly from that of RF4/PPR782, exhibiting several amino acid substitutions and a 14‐amino acid deletion. RF4/PPR782 has been reported to trigger RNA degradation of the *WA352* mRNA, without any evidence of associated cleavage (Kazama and Toriyama 2014). The distance between the predicted binding site (BS) and the 3′ end of the major cleavage site is 79 nucleotides for the *orf312* RNA in TAR (Figure 6), which is slightly longer than distances reported for other mitochondrial genes: 31 nt in *atp6*‐*orf79* for BT‐CMS rice (Kazama et al. 2008), 65 nt or 1 nt in *orf279* for *Triticum timopheevii* ‐type CMS (Melonek et al. 2021), 48 nt or more in *orf291*, 21 nt or 52 nt in *ccmB*, 69 nt and 68 nt for *nad9* and *cox3*, respectively, in *Arabidopsis thaliana* (Jonietz et al. 2010; Fujii et al. 2016; Stoll et al. 2017).

The *PPR796*‐transgenic plants, *PPR796*#1 and *PPR79*6#2, showed seed setting rates of 91.1% and 44.3%, respectively (Table S3), indicating that the *PPR796* has the potential for full male fertility recovery. We must note, however, that the amount of intact 1.15‐kb *orf312* mRNAs remains quite high in the *PPR796*‐transgenic plants (Figure 4). If translated, such a high level of intact *orf312* mRNAs should significantly interfere with male fertility. Our results suggest that PPR796 could possess an additional role in blocking the translation of the *orf312* RNA, similar to the mechanism reported for Ogura CMS in rapeseed, where the Rf protein, PPR‐B/Rfo, binds to the beginning of the CMS‐causing gene *orf138* and blocks the translation elongation, resulting in reduced accumulation of the ORF138 protein (Wang et al. 2021). Efforts are being made to raise antibodies against the recombinant ORF312 to measure its steady‐state levels in both TA‐CMS and *PPR796*‐transgenic plants.

RNA cleavage of an *orf312*‐like CMS‐causing gene has also been reported for *orf352*, a sequence variant of *WA352* with a five‐nucleotide polymorphism, in the study of fertility restoration of RT102‐CMS (Okazaki et al. 2013; Omukai et al. 2021). The *orf352‐containing* RNA is cleaved at 19–69 nt upstream of the stop codon of *orf352* mediated by an unidentified *Rf* factor (Okazaki et al. 2013). The nucleotide sequences around the cleavage sites of the *orf312* RNA and *orf352* RNA were similar and shared close cleavage sites (Figure 6, Figure S7). This suggests that the restorer line for RT102‐CMS might carry a *PPR796* allele whose encoded protein has a binding site identical to that of RFta/PPR796.

The amino acid sequence of the C‐terminal region of WA352 has been reported to interact with COX11 and to induce a burst of reactive oxygen species (Luo et al. 2013). This sequence, named the cs1‐encoded region, is well conserved across several mitochondrial CMS‐causing and CMS‐related proteins, including WA352a, WA352b, WA352c, WA314, ORF356, ORF367, ORF314, ORF310, ORF276, ORF284a, and ORF284b (Tang et al. 2017). A nucleotide sequence highly homologous to the cs1 sequence was also found in *orf312* of TA‐CMS and *orf352* of RT102‐CMS (Figure S7). The predicted RNA binding site of RF4/PPR782 in the WA352‐containing RNA is 5′‐GGUUGCACCAAAUGCUCG‐3′, which is also found in the *orf352*‐containing RNA in RT102‐CMS. This site also corresponds to the target site of RFta/PPR796 in the *orf312* RNA, differing by two nucleotide substitutions within the 18‐nucleotide sequence (Figure S7). Moreover, RFta/PPR796 for TA‐CMS and RF4/PPR782 for WA‐CMS showed 98% amino acid identity, except for RfCTD (Figure S2). Hence, the nucleotide‐binding site identified in this study would be a potential common target of RF4‐like PPR proteins. Molecular cloning of the RF4‐like PPR gene responsible for the cleavage of the *orf352*‐containing RNA and comparison of the binding affinity of each RF4‐like PPR protein against the respective BS would facilitate our understanding of the coevolution between the mitochondrial cs1 sequences and RF4‐like PPR genes.

## Author Contributions

K.T. and A.T. designed the project. A.T. mainly performed experiments under the instruction of K.I., T.K., and K.T. Y.I. constructed the binary vector. H.M. and A.T. performed RNA gel electrophoresis mobility shift assay. H.I. and T.A. analyzed WGS data. A.T., T.K., and K.T. wrote the manuscript.

## Conflicts of Interest

The authors declare no conflicts of interest.

## Supporting information


**Figure S1.** Expression profile of *orf312* in fertile and sterile recombinant plants detected using RNA‐gel blot analysis. RNA was extracted from the flowering anthers of BC_3_F_5_ lines 66_18_16, 66_18_40, 66_36_2 and 66_36_4, whose genotypes are shown in Table 1, along with T65, TAA and TAR. Methylene blue‐stained rRNA was used as a loading control.
**Figure S2.** Comparison of the amino acid sequences of RF proteins among RF4 (GenBank accession no. AIC74551.1), RF1a (accession no. BAC77665.2) and RFta/PPR796. A mitochondrial targeting signal peptide predicted by MitoFates is underlined. PPR motifs were predicted using PPRFinder and are highlighted in yellow and green. Amino acids RF4 and RF1a, which are distinct from RFta/PPR796, are indicated in red. The blue line encloses the RfCTD (Huynh et al. 2023). The amino acids of RF1a that are distinct from RFta within the RfCTD are indicated in light blue.
**Figure S3.** The genomic fragment of PPR genes used for complementation test. These fragments were amplified using PCR with *Sal*I or *Bam*HI site.
**Figure S4.** Anther phenotype and seed setting rates of *PPR796*‐transgenic lines, T65, TAA and TAR. White arrows indicate the dehiscent anthers. The details of seed setting rates are listed in Table S5. Bars = 1 mm.
**Figure S5.** Anther phenotype and seed setting rates of (a) *PPR782*, (b) *PPR762* and (c) *PPR683*‐transgenic lines. White arrows indicate the dehiscent anthers. The details of seed setting rates are listed in Table S5. Bars = 1 mm.
**Figure S6.** Anther phenotype and seed setting rates of (a) *PPR777* and (b) *PPR794*‐transgenic lines. White arrows indicate the dehiscent anthers. The details of seed setting rates are listed in Table S5. Bars = 1 mm.
**Figure S7.** Nucleotide sequences of cs1 region in *WA352* of WA‐CMS (GenBank accession no. JX131325.1), *orf352* of RT102‐CMS (GenBank accession no. AP012528) and *orf312* of TA‐CMS (GenBank accession no. LC592696.1). Predicted binding sites (BS) of RF4 for *WA35*2 and RFta/PPR796 for *orf312* are highlighted in green. Distinct nucleotides within BS are indicated in red. Detected 3′‐terminal ends in the RT102‐restorer line (Okazaki et al. 2013) and in the *PPR796*‐transgenic plant are highlighted in yellow.
**Table S1.** Primers of markers in chromosome 10 used for fine mapping of *Rfta*.
**Table S2.** Primers used for cloning genomic fragments, RT‐PCR, RACE and REMSA.
**Table S3.** Seed setting rates of recombinant lines in Table 1.
**Table S4.** Position and annotation of genes predicted by MEGANTE in *Rfta* candidate region.
**Table S5.** Seed setting rates and anther phenotypes of *PPR*‐transgenic TAA lines.
**Table S6.** Predicted sequences of the PPR796‐binding site (BS) on *orf31*2 transcript.
**Table S7.** Number of clones in RACE analysis.

## Data Availability

All relevant data can be found within the article and Figures S1–S7 and Tables S1–S7. The DNA sequence has been deposited in DDBJ with accession number LC868044.
