# Supplementary material for: A Pentatricopeptide Repeat Protein Restores Fertility in Tadukan‐Type Cytoplasmic Male Sterile Rice via the Cleavage of the Mitochondrial orf312 RNA
Source: Physiol Plant. 2025 May 29;177(3):e70308. doi: 10.1111/ppl.70308 (PMC12123059; doi:10.1111/ppl.70308)
Supplement: Supplementary file 1 — Figure S1. Expression profile of orf312 in fertile and sterile recombinant plants detected using RNA‐gel blot analysis. RNA was extracted from the flowering anthers of BC3F5 lines 66_18_16, 66_18_40, 66_36_2 and 66_36_4, whose genotypes are shown in Table 1, along with T65, TAA and TAR. Methylene blue‐stained rRNA was used as a loading control. Figure S2. Comparison of the amino acid sequences of RF proteins among RF4 (GenBank accession no. AIC74551.1), RF1a (accession no. BAC77665.2) and RFta/PPR796. A mitochondrial targeting signal peptide predicted by MitoFates is underlined. PPR motifs were predicted using PPRFinder and are highlighted in yellow and green. Amino acids RF4 and RF1a, which are distinct from RFta/PPR796, are indicated in red. The blue line encloses the RfCTD (Huynh et al. 2023). The amino acids of RF1a that are distinct from RFta within the RfCTD are indicated in light blue. Figure S3. The genomic fragment of PPR genes used for complementation test. These fragments were amplified using PCR with SalI or BamHI site. Figure S4. Anther phenotype and seed setting rates of PPR796‐transgenic lines, T65, TAA and TAR. White arrows indicate the dehiscent anthers. The details of seed setting rates are listed in Table S5. Bars = 1 mm. Figure S5. Anther phenotype and seed setting rates of (a) PPR782, (b) PPR762 and (c) PPR683‐transgenic lines. White arrows indicate the dehiscent anthers. The details of seed setting rates are listed in Table S5. Bars = 1 mm. Figure S6. Anther phenotype and seed setting rates of (a) PPR777 and (b) PPR794‐transgenic lines. White arrows indicate the dehiscent anthers. The details of seed setting rates are listed in Table S5. Bars = 1 mm. Figure S7. Nucleotide sequences of cs1 region in WA352 of WA‐CMS (GenBank accession no. JX131325.1), orf352 of RT102‐CMS (GenBank accession no. AP012528) and orf312 of TA‐CMS (GenBank accession no. LC592696.1). Predicted binding sites (BS) of RF4 for WA352 and RFta/PPR796 for orf312 are highlighted [file PPL-177-e70308-s001.pdf]

Supporting information

A pentatricopeptide repeat protein restores fertility in Tadukan-type cytoplasmic male sterile rice via the cleavage of the mitochondrial *orf312* RNA

Ayumu Takatsuka[1], Yuko Iwai[1], Hakim Mireau[2], Tomohiko Kazama[3], Hiroyuki Ichida[4], Tomoko Abe[4], Keisuke Igarashi[1], Kinya Toriyama[1]

1 Graduate School of Agricultural Science, Tohoku University,

468-1 Aramaki Aza Aoba, Aoba-ku, Sendai, Miyagi, 980-8572, Japan

2 Université Paris-Saclay, INRAE, AgroParisTech, Institute Jean-Pierre Bourgin for Plant Sciences (IJPB), 78000, Versailles, France

3 Graduate School of Agriculture, Kyushu University, Fukuoka, Fukuoka 819-0395, Japan

4 RIKEN Nishina Center for Accelerator-Based Science, Wako, Saitama 351-0198, Japan

***Correspondence**

Kinya Toriyama

E-mail: torikin@tohoku.ac.jp

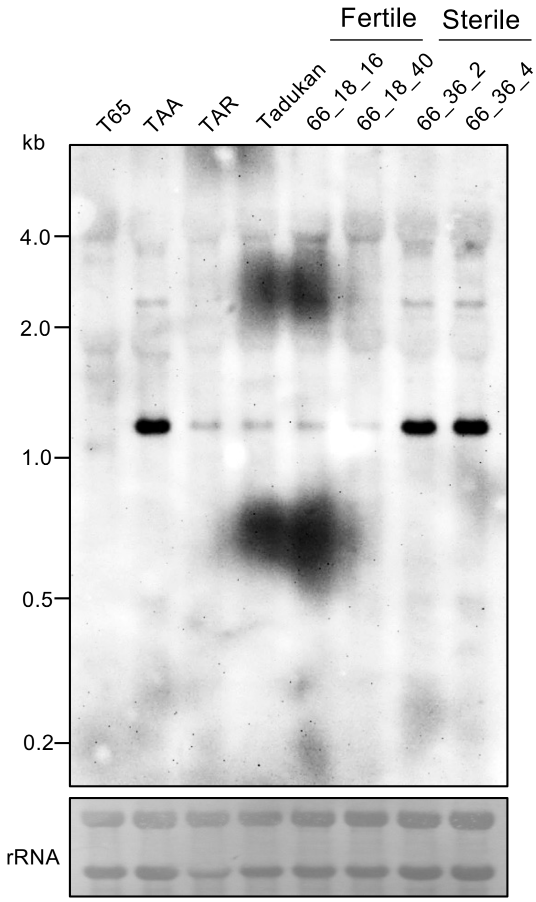

Fig. S1 Fig. S1 Expression profile of *orf312* in fertile and sterile recombinant plants detected using RNA-gel blot analysis.

RNA was extracted from the flowering anthers of BC_3F_5 lines 66_18_16, 66_18_40, 66_36_2, and 66_36_4, whose genotypes are shown in Table 1, along with T65, TAA, and TAR. Methylene blue-stained rRNA was used as a loading control.

```
WA_RF4/PPR782     MARRVPTRPRGGGGGGVPRSEGSIQGRGGRAGGSGAEDARHVFDELLRRGRGASIYGLNR
TA_RFta/PPR796    MARRVPTRPRGGGGGGVPRSEGSIQGRGGRAGGSGAEDARHVFDELLRRGRGASIYGLNR
BT_RF1a/PPR791    -----MARRAASRAVGALRSDGSIQGRGGRAGGSGAEDARHVFDELLRRGRGASIYGLNR
                       :*  .. .  *. **:***************************************

                                                                PPR 1
WA_RF4/PPR782     ALADVARHSPAAAVSRYNRMARAGAGKVTPTVHTYAILIGCCCRAGRLDLGFAALGNVVK
TA_RFta/PPR796    ALADVARHSPAAAVSRYNRMARAGAGKVTPTVHTYAILIGCCCRAGRLDLGFAALGNVVK
BT_RF1a/PPR791    ALADVARDSPAAAVSRYNRMARAGADEVTPDLCTYGILIGCCCRAGRLDLGFAALGNVIK
                  *******.*****************.:*** : **.**********************:*

                                          PPR 2
WA_RF4/PPR782     KGFRVDAITFTPLLKGLCADKRTSDAMDIVLRRMTELGCIPDVFSYNILLKGLCDENRSQ
TA_RFta/PPR796    KGFRVEAITFTPLLKGLCADKRTSDAMDIVLRRMTELGCIPDVFSYNILLKGLCDENRSQ
BT_RF1a/PPR791    KGFRVDAIAFTPLLKGLCADKRTSDAMDIVLRRMTELGCIPNVFSYNILLKGLCDENRSQ
                  *****:**:********************************:******************
                  PPR 3                                     PPR 4
WA_RF4/PPR782     EALELLHMMADDRGGGSPPDVVSYNTVLNGFFKEGDSDKAYSTYHEMLDRGILPDVVTYS
TA_RFta/PPR796    EALELLHMMADDRGGGSPPDVVSYNTVLNGFFKEGDSDKAYSTYHEMLDRGILPDVVTYS
BT_RF1a/PPR791    EALELLHMMADDRGGGSPPDVVSYTTVINGFFKEGDSDKAYSTYHEMLDRGILPDVVTYN
                  ************************.**:*******************************.

                      PPR 5                                             PPR 6
WA_RF4/PPR782     SIIAALCKAQAMDKAMEVLTTMVKNGVMPDCMTYTSIMHGYCSSGQPKEAIGFLKKMRSD
TA_RFta/PPR796    SIIAALCKAQAMDKAMEVLTTMVKNGVMPDCMTYTSIMHGYCSSGQPKEAIGFLKKMRSD
BT_RF1a/PPR791    SIIAALCKAQAMDKAMEVLNTMVKNGVMPDCMTYNSILHGYCSSGQPKEAIGFLKKMRSD
                  *******************.**************.**:**********************

                                         PPR 7
WA_RF4/PPR782     GVKPNVFTYRSLMNYLCKNGRSTEARKIFDSMTKRGLEPDIATYRTLLQGYATKGALVEM
TA_RFta/PPR796    GVEPNVFTYRSLMNYLCKNGRSTEARKIFDSMTKRGLEPDIATYRTLLQGYATKGALVEM
BT_RF1a/PPR791    GVEPDVVTYSLLMDYLCKNGRCMEARKIFDSMTKRGLKPEITTYGTLLQGYATKGALVEM
                  **:*:*.**  **:*******. **************:*:*:** ***************
                  PPR 8                        PPR 9
WA_RF4/PPR782     HALLDLMVRNGIQPDHHVFNILICAYAKQEKVDQAMLVFSKMRQHGLNPNVVTYGTVIDV
TA_RFta/PPR796    HALLDLMVRNGIQPDHHVFNILICAYAKQEKVDQAMLVFSKMRQHGLNPNVVTYGTVIDV
BT_RF1a/PPR791    HGLLDLMVRNGIHPDHYVFSILICAYAKQGKVDQAMLVFSKMRQQGLNPNAVTYGAVIGI
                  *.**********:***:**.********* **************:*****.****:**.:

                          PPR 10                             PPR 11
WA_RF4/PPR782     LCKSGSVDDAMLYFEQMIDEGLTPNIIVYTSLIHGLCTYDKWEKAEELFFKMLDSGICPN
TA_RFta/PPR796    LCKSGSVDDAMLYFEQMIDEGLTPNIIVYTSLIHGLCTYDKWEKAEELFFKMLDSGICPN
BT_RF1a/PPR791    LCKSGRVEDAMLYFEQMIDEGLSPGNIVYNSLIHGLCTCNKWERAEELILEMLDRGICLN
                  ***** *:**************:*. ***.******** :***:****:::*** *** *

                                    PPR 12                           PPR 13
WA_RF4/PPR782     TVFFSSIISNLCKEGRVIESEKLFDLMVRIGVKPNVITYNTLIDGCCLGGKMDEAMKLLS
TA_RFta/PPR796    TVFFSSIISNLCKEGRVIESEKLFDLMVRIGVKPNVITYNTLIDGCCLAGKMDEAMKLLS
BT_RF1a/PPR791    TIFFNSIIDSHCKEGRVIESEKLFELMVRIGVKPNVITYNTLINGYCLAGKMDEAMKLLS
                  *:**.***.. *************:******************:* **.***********

                                         PPR 14
WA_RF4/PPR782     GMVSVGLKPNTVTYSTLINGYCKISRMEDALVLFKEMESSGVSPDIITYNIILQGLFQTR
TA_RFta/PPR796    GMVSVGLKPNTVTYSTLINGYCKISRMEDALVLFKEMESSGVSPDIITYNIILQGLFQTR
BT_RF1a/PPR791    GMVSVGLKPNTVTYSTLINGYCKISRMEDALVLFKEMESSGVSPDIITYNIILQGLFQTR
                  ************************************************************

                   PPR 15                               PPR 16
WA_RF4/PPR782     RTAAAKELYVSITKSGTQLELSTYNIILHGLCKNNLTDEALRMFQNLCLTDLQLETRTFN
TA_RFta/PPR796    RTAAAKELYVGITESGTQLELSTYNIILHGLCKNNLTDEALRMFQNLCLTDLQLETRTFN
BT_RF1a/PPR791    RTAAAKELYVRITESGTQIELSTYNIILHGLCKNKLTDDALQMFQNLCLMDLKLEARTFN
                  ********** **:****:***************:***:**:******* **:**:****

                             PPR 17                                PPR 18
WA_RF4/PPR782     IMIGALLKCGRMDEAKDLFAAHSANGLVPDVWTYSLMAENLIEQGSLEELDDLFLSMEEN
TA_RFta/PPR796    IMIGALLKVGRNDEAKDLFAALSANGLVPDVRTYSLMAENLIEQGLLEELDDLFLSMEEN
BT_RF1a/PPR791    IMIDALLKVGRNDEAKDLFVAFSSNGLVPNYWTYRLMAENIIGQGLLEELDQLFLSMEDN
                  ***.**** ** *******.* *:*****:  ** *****:* ** *****:******:*

WA_RF4/PPR782     GCSADSRMLNSIVRKLLQRGDITRAGTYLFMIDEKHFSLEASTASFLLESSPIVWEQISR
TA_RFta/PPR796    GCTANSRMLNSIVRKLLQRGDITRAGTYLFMIDEKHFSLEASTASLFLDLLSGGKYQEYH
BT_RF1a/PPR791    GCTVDSGMLNFIVRELLQRGEITRAGTYLSMIDEKHFSLEASTASLFIDLLSGGKYQEYY
                  **:.:* *** ***:*****:******** ***************::::  .    *

WA_RF4/PPR782     IS--------------     RfCTD
TA_RFta/PPR796    [illegible]KYKSFIESLSC
BT_RF1a/PPR791    RFLPEKYKSFIESLSC
```

Fig. S2 Comparison of the amino acid sequences of RF proteins among RF4 (GenBank accession no. AIC74551.1), RF1a (accession no. BAC77665.2) and RFta/PPR796.

A mitochondrial targeting signal peptide predicted by MitoFates is underlined. PPR motifs were predicted using PPRFinder and are highlighted in yellow and green. Amino acids RF4 and RF1a, which are distinct from RFta/PPR796, are indicated in red. The blue line encloses the RfCTD (Huynh et al., 2023). The amino acids of RF1a that are distinct from RFta within the RfCTD are indicated in light blue.

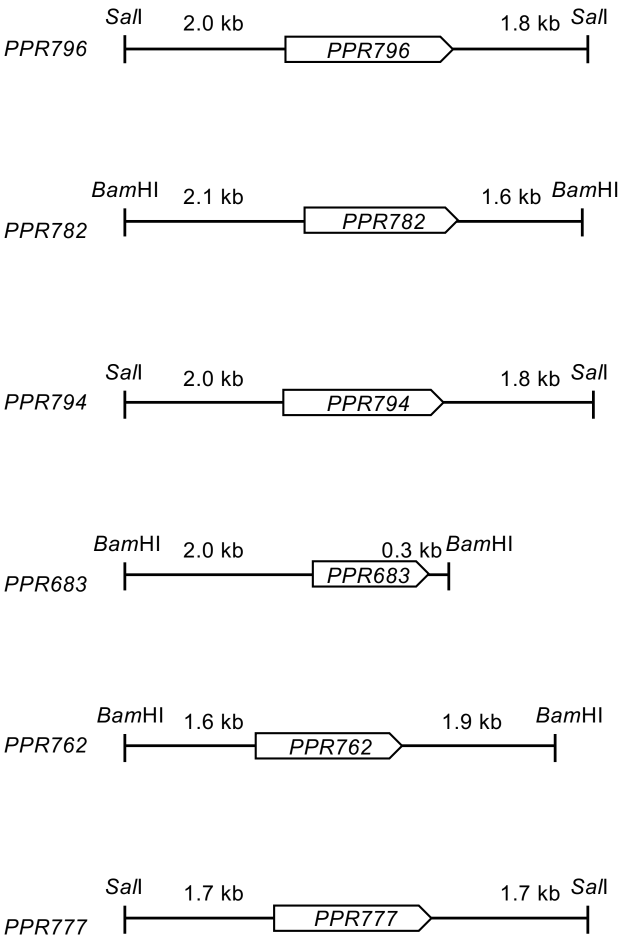

Fig. S3 The genomic fragment of PPR genes used for complementation test. These fragments were amplified using PCR with *Sal*I *or Bam*HI site.

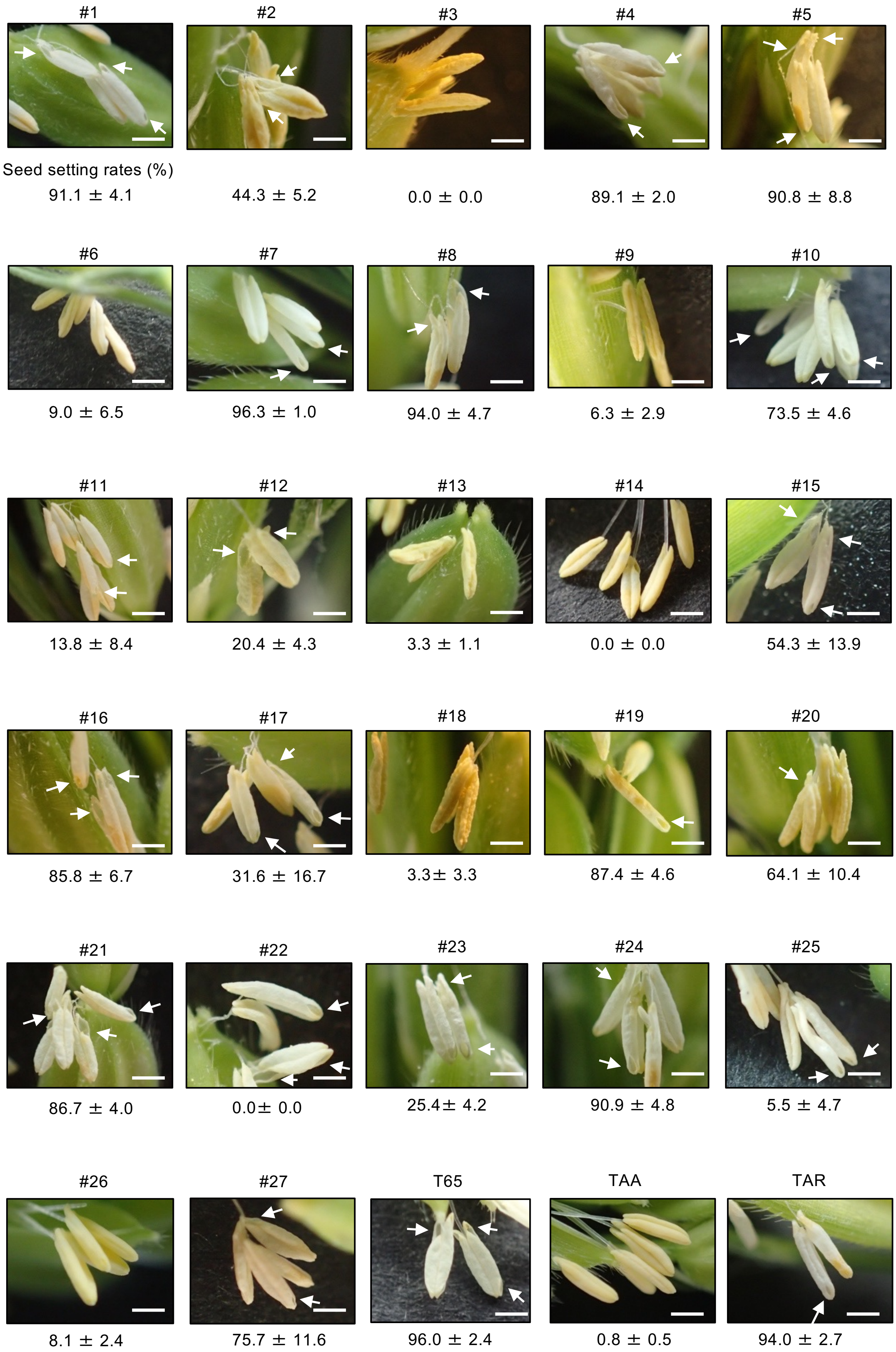

Fig. S4 Anther phenotype and seed setting rates of PPR796-transgenic lines, T65, TAA, and TAR. White arrows indicate the dehiscent anthers. The details of seed-setting rates are listed in Table S5. Bars = 1 mm.

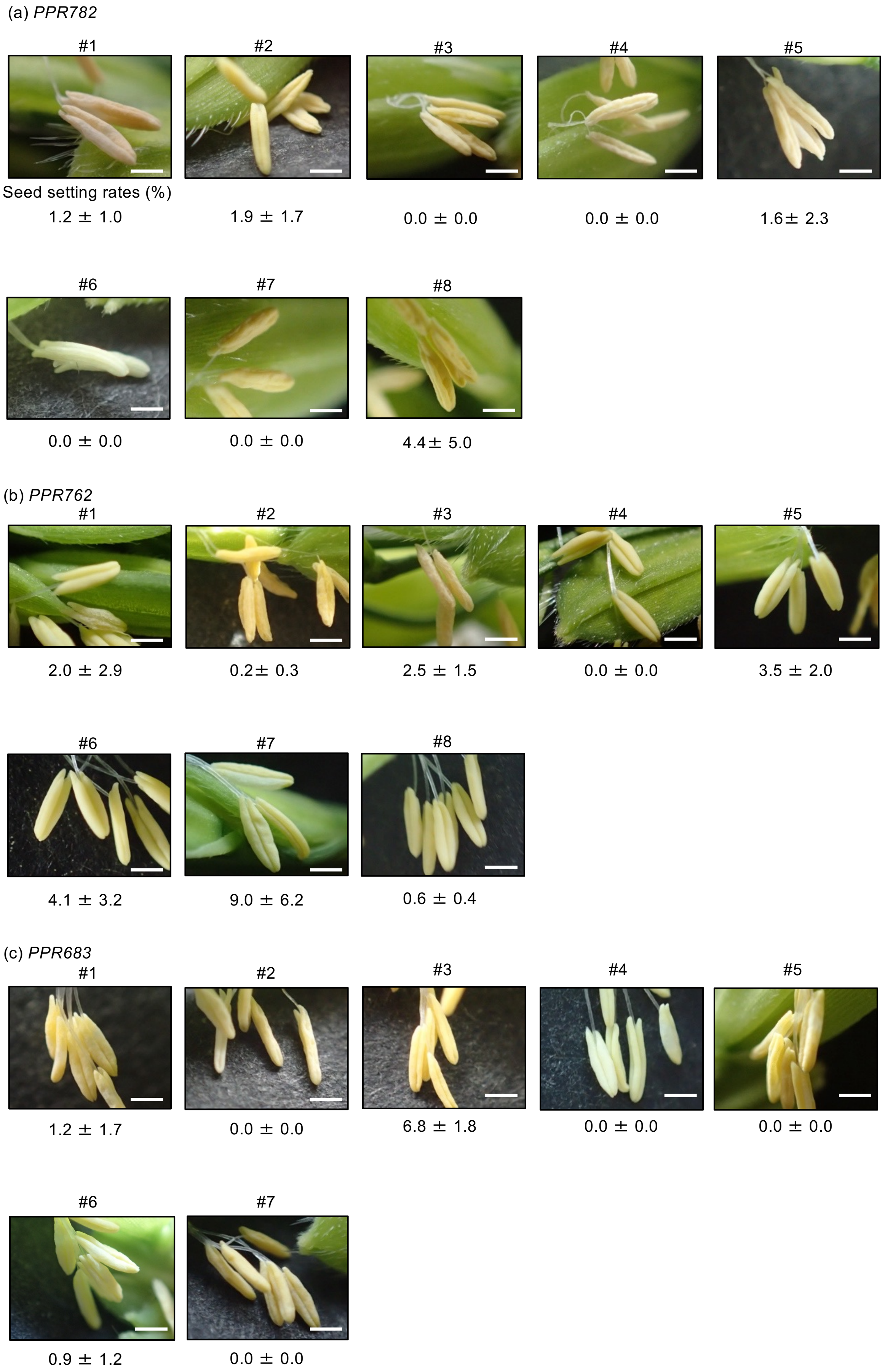

Fig. S5 Anther phenotype and seed setting rates of (a) PPR782, (b) PPR762, and (c) PPR683-transgenic lines. White arrows indicate the dehiscent anthers. The details of seed setting rates are listed in Table S5. Bars = 1 mm.

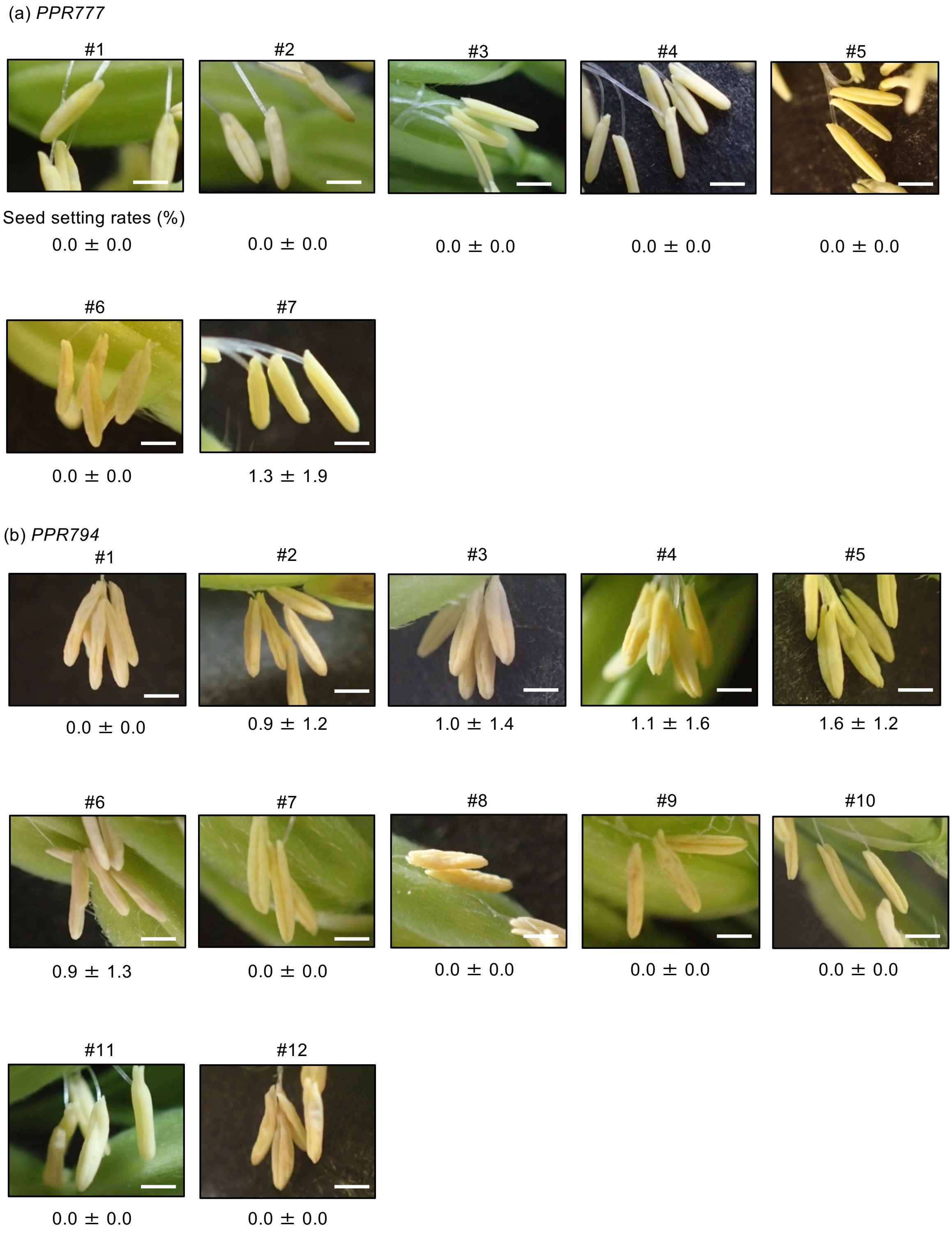

Fig. S6 Anther phenotype and seed setting rates of (a) PPR777 and (b) PPR794-transgenic lines. White arrows indicate the dehiscent anthers. The details of seed-setting rates are listed in Table S5. Bars = 1 mm.

```
cs1_WA352_WA        TCGGTCAATCAACCGCTTCCAGGGGAGCAAGCTATGCCTCCCGCTCTTCCCGTTATGCAG
cs1_orf352_RT102    TCGGTCAATCAACCGCTTCCAGGGGAGCAAGCTATGCCTCCCGCTCTTCCCGTTATGCAG
cs1_orf312_TA       TCGGTCAATCAACCGCTTCCAGGGGAGCAAGCTATGCCTCCCGCTCTTCCCGTTATGCAG
                    ************************************************************

cs1_WA352_WA        GAAGCTGCTAATCGGTCTCCGCCCTACGCGCCCTACCCGTATCCAGTTGACGAGATAATA
cs1_orf352_RT102    GAAGCTGCTAATCGGGCTCCGCCCTACGCGCCCTACCCGTATCCAGTTGACGAGATAATA
cs1_orf312_TA       GAAGCTGCTAATCGGGCTCCGCCCTACGCGCCCTACCCGTATCCAGTTGACGAGATAATA
                    *************** ********************************************

cs1_WA352_WA        GGAGGGGATAGCGTGCAATCCATTCAAAGAAGACTTTTGGGGACTAATTGGAATCCTTCC
cs1_orf352_RT102    GGAGGGGATAGCGTGCAATCCATTCAAAGAAGACTTTTGGGGACTAATTGGAATCCTTCC
cs1_orf312_TA       GGAGGGGATAGCGTAGAATCCATTCAAAGGAGGCTGTTGGGGACTAATTGGAATCCTTCA
                    **************  ************* ** ** *********************** 

cs1_WA352_WA        GCCCATGACATGCAAATGTCCCGGATTCAAGCGGAGGATCTATTTGAACTGAAAGTGGAA
cs1_orf352_RT102    GCCCATGACATAAAAATGTCCCGGATTCAAGCGGAGGATCTATTTGAACTGAAAGTGGAA
cs1_orf312_TA       GCCCATGACATAAGAATGGCCCGGATGCAAGCCGAAGACCTTTTTGAGGTGAAGGTTGAC
                    ***********   **** ******* ***** ** ** ** *****  **** ** ** 

cs1_WA352_WA        ATCATAAGAAAGATGGCGGGCCTGCATCCAAGTGGCGATTGGATGGGATGGGGCGCGCGG
cs1_orf352_RT102    ATCATAAGAAAGATGGCGGGCCTGCATCCAAGTGGCGATTGGATGGGATGGGGCGCGCGG
cs1_orf312_TA       ATAATCCGGAAGATGGCGGGCCTGCATCCAAGTGGCGATTGGATGGGATGGGGGGCGCGG
                    ** **  * ******************************************** ******

                                                             Predicted BS in RNA
cs1_WA352_WA        GCCTTGGACAACCCCCGTACGGCCACTGGCGAGGAAGACTTGGCTAGGTTGCACCAAATG
cs1_orf352_RT102    GCCTTGGACAACCCCCGTACGGCCACTGGCGAGGAAGACTTGGCTAGGTTGCACCAAATG
cs1_orf312_TA       GCCCTGGACAACCCCCGTACGGCCACTGGTGAGGAAGACTTGGCTAGGTTGCATCAAATG
                    *** ************************* *********************** ******

                                        Cleavage sites in RNA
cs1_WA352_WA        CTCGACGACCTACAGAGCCGGAATGAGCAATCAGCTACCTTCTGGCGCTTGGTCGAAAGA
cs1_orf352_RT102    CTCGACGACCTACAGAGCCGGAATGAGCAATCAGCTACCTTCTGGCGCTTGGTCGAAAGA
cs1_orf312_TA       CTTGACGACCTACAGAGCGGGAATTCGCAATCTGCAACCTTCTGGCTCTTGGTCGAAAGA
                    ** *************** *****  ****** ** ********** *************

cs1_WA352_WA        GTCCGCTTACGGGCGGATGAGGATCAAAACTCAGCCTCCTAG
cs1_orf352_RT102    GTCCGCTTACGGGCGGATGAGGATCAAAACTCAGCCTCCTAG
cs1_orf312_TA       GTCCGCTTACGGGCGGGTGAGGATCAAAACTCCGCTTCCTAG
                    **************** *************** ** ******
```

Fig. S7 Nucleotide sequences of cs1 region in *WA352* of WA-CMS (GenBank accession no. JX131325.1), *orf352* of RT102-CMS (GenBank accession no. AP012528) and *orf312* of TA-CMS (GenBank accession no. LC592696.1).
Predicted binding sites (BS) of RF4 for *WA352* and RFta/PPR796 for *orf312* are highlighted in green. Distinct nucleotides within BS are indicated in red. Detected 3'-terminal ends in the RT102-restorer line (Okazaki et al., 2013) and in the *PPR796*-transgenic plant are highlighted in yellow.

Table S1 Primers of markers in chromosome 10 used for fine mapping of *Rfta*

Marker name	Forward sequence (5'->3')	Reverse sequence (5'->3')
SSR1041	AATCGAATCTGGATATCTTG	CTTCTACCTAGCTACCGAGA
SSR10081	ATGCTCTCAAGTGTGTCAAGG	AACCTCTGGAGTATGTGTAGTGC
SSR10085	GGCCTGGAGCTAGATAGAGTTGG	CGTCAACTGCAAAGTCCAAAGG
SSR H10004	CAACTCCCCCGAGAAGAAT	CCACCATCTCCTCTTTCACC
SSR H10023	GGCGAAGTCGAAGTCCAG	GCCTTTTCCAAACCGACTTT
SSR H10040	GGCCACCACATGTAAGCAC	TCCATGTGTGAGTGATCTTTTTG
SSR H10045	GACCACTTCGTTCGATTCCT	ATGCGACAGCCAAACAAAC
SSR H10070	GGGGTCCAGCTCGAGTATC	GCAAAACGCAAAACAAGCTC
TTRf-indel19,120	GTGTGATCACACTCTTGTGC	TAGCATCGAGGACGTACGTC
TTRf-Indel19,134	TTTGTTTTACTGTTCATCGG	GCTAATCACTTGTTGTGACG
KNJ8-indel759	AACCGATCGATCTCACGTTCT	AAATCAAAGCCGGTGAAATG
SSR1061	TCCGTATCCTAGTCGCGATC	CGCCGTCATGACTCATACTC
SSR1062	CTCTCTCTCCCCACCCAATC	GAGGGAGGAGGAGGTGTAGG
SSR1065	ATTTCCCACACATCTCGCTG	GTGTCGCCGGTCAAGAAC
SSR1069	GAGAGAACCTGGTGGTGGAG	AGTGGTAGAAGATCCGAGATCG

Table S2 Primers used for cloning genomic fragments, RT-PCR, RACE, and REMSA

Experiment	Name	Sequence (5' > 3')	Purpose	Remarks
	TA-796-F3	CGTTCAGTACTGAAGACCCG		
	TA-796-R1	ATGCGTGTGATTACCGTGCG		
	TA-782-F1	AAAGGATCCCAGGTGAGCCTCTTCTTGCT		*Bam*HI site
	TA-782-R1	AAAGGATCCTCGATAGTTAATAGTTAAGG		
	TA-794-F1	AAAGTCGACAAGGGCCGAGCATGCCACTG		*Sal* I site
	TA-794-R3	AAAGTCGACCAGCGACGTGGGCGATTACG	1st-PCR	
	TA-683-F	AAAGGATCCGGGGAAACTTGGTAATTATA		*Bam*HI site
	TA-683-R	AAAGGATCCGATCAATATATACCTACTGC		
Cloning of genomic fragment	TA-762-F	AAAGGATCCGATTCACGAATTTGATTAGT		*Bam*HI site
	TA-762-R	AAAGGATCCGAAGTCGACCGGCACAGCCG		
	TA-777-F	AAAGTCGACATATTGCTATATTACAGGAC		*Sal* I site
	TA-777-R	AAAGTCGACCAAGGTACCACGCTTGCCGC		
	TA-796-F	AAAGTCGACATGCTCTGCATCGGTGGCAA		*Sal* I site
	TA-796-R	AAAGTCGACGGAACAAGCCTGCACAATCC		
	TA-782-F	AAAGGATCCGGGTACTCTCAGCCCGAGCC	2nd-nested PCR	*Bam*HI site
	TA-782-R1	AAAGGATCCTCGATAGTTAATAGTTAAGG		
	TA-794-F	AAAGTCGACCCCACACCGCCACGGCGTCA		*Sal* I site
	TA-794-R	AAAGTCGACCTTTCATGTGGCTTCAAGCG		
	Oligo d(T)	GAGAGAACTAGTCTCGAGTTTTTTTTTTTTTTTTTT	Reverse transcription	
	PPR407 F1hs	GAAGCTAGAAAGATTTCCA		
	PPR407 R1	TCTATAACACTTCCATTTATC		
	PPR506 F1hs	ACAGAGGCTATTAAATTTCCAG		
	PPR506 R1	GACTAACACCATTGCTCAC		
	PPR762 F1	GTATGGAGCAGTTATAGGCA		
	PPR762 R1hs	AACTGAGAACATGCTGAAG		
	PPR777 F1hs	AGCAATGAAGTTACTTTATG		
RT-PCR	PPR777 R1	CTTCATCCATTCTTCCACA	PCR	
	PPR782 F1	CAAAAGAGGCTATTGGAACA		
	PPR782 R1hs	TAGCCATTAATCAAGGTACC		
	PPR794 F1hs	ATAATAGTATCGTGCATAGG		
	PPR794 R1	TTGTCAAAGATACAGAGACT		
	PPR683_F2hs	CCTATGTTTGATGGATTCGA		
	PPR683_R2	TTTAGCATGCCAGAGTCAA		
	PPR796_F1	TCTTCAAAGAGGGGGATTCA		
	PPR796_R1hs	AAGTAACAGTATTAGGTTTCGAC		
RACE	IF-orf312_3RACE_F	GATTACGCCAAGCTTATGGCATCAACGACTCCTTCCG	3' RACE	Identical part to pRACE
	PPR762-777-782-796GW5	CAAAAAAGCAGGCTTAGCCTCGATCTACGGCTTGAACCGC	1st-PCR for BP reaction	
	PPR683-794-796GW3	CAAGAAAGCTGGGTCTAGCAGCTCAAAGATTCTATAAAG		Identical part for nested-PCR
	GW5	GGGGACAAGTTGGTACAAAAAAGCAGGCT	2nd-PCR for BP reaction	
	GW3	GGGGACCACTTTGTACAAGAAAGCTGGGT		
	GMSI1probe_T7F	ATGCTAATACGACTCACTATAGAGCCCATGACATAAGAATGG		3' BS_probe 1
	GMSI1probe_R	CCAAGTCTTCCTCACCAGTG		
REMSA	GMSI2probe_T7F	ATGCTAATACGACTCACTATAGACATAATCCGGAAGATGGC		3' BS_probe 2
	GMSI2probe_R	GGTTGCAGATTGCGAATTCC		
	GMSI3probe_T7F	ATGCTAATACGACTCACTATAGTGCATCCAAGTGGCGATTGG	RT templates	3' BS_probe 3
	GMSI3/4probe_R	AGGAAGCGGAGTTTTGATCC		3' BS_probe 3, 4
	GMSI4probe_T7F	ATGCTAATACGACTCACTATAGTACGGCCACTGGTGAGGAAG		3' BS_probe 4
	GMSI5probe_T7F	ATGCTAATACGACTCACTATAGTACAGAGCGGGAATTCGC		3' BS_probe 5
	GMSI5probe_R	TTAGTATAGATGGATACCTACAGCG		

Table S3 Seed setting rates of recombinant lines in Table 1

Generation	Plant No.	Filled spiklets / Total spikelets			Ave. (%)	(±) S.D.
		Panicle 1	Panicle 2	Panicle 3		
BC_3F_4	66_5	0/45	0/50	0/63	0.0	0.0
	66_18	58/63	54/67	54/60	87.6	5.0
	66_36	5/90	0/81	4/102	3.2	2.3
BC_3F_5	66_18_16	40/69	75/87	82/88	79.1	15.2
	66_18_40	54/67	38/58	68/97	72.1	6.3
	66_36_2	1/79	0/75	0/59	0.4	0.6
	66_36_4	0/48	0/59	0/60	0.0	0.0
	TAA	3/97	0/72	1/83	1.4	1.6
	TAR	115/120	102/108	88/97	93.7	2.6
	Tadukan	122/134	95/98	130/139	93.8	2.4

Table S4 Position and annotation of genes predicted by MEGANTE in *Rfta* candidate region

Gene ID	Description	Start	End	Orientation	Evidence
Gene_1.1	Kiwellin	340	828	-	None
		340	828		
Gene_2.1	Hypothetical protein	3944	4168	-	None
		3944	4168		
Gene_3.1	Linalool synthase, chloroplastic	11032	14447	-	Supported by EST or cDNA
		14327	14447		
		14129	14326		
		13101	13392		
		12394	12769		
		11516	11734		
		11224	11380		
		11032	11223		
Gene_4.1	Kiwellin	26048	27278	+	Supported by EST or cDNA
		26048	26471		
		26472	27191		
		27192	27278		
Gene_5.1	Harpin inducing protein	30521	31643	+	Supported by EST or cDNA
		30521	30649		
		30650	31450		
		31451	31643		
Gene_6.1	Hypothetical protein	33294	35532	+	Supported by EST or cDNA
		33294	33393		
		33394	33469		
		34718	34755		
		35188	35301		
		35302	35532		
Gene_7.1	CDT1a protein	38558	43262	+	Supported by EST or cDNA
		38558	38727		
		39466	39471		
		39472	39833		
		39917	40036		
		40115	40343		
		40554	41213		
		41785	41986		
		42082	42172		
		42341	42398		
		42399	42993		
		43236	43262		
Gene_7.2	CDT1a protein	38558	43262	+	Supported by EST or cDNA
		38558	38727		
		39457	39471		
		39472	39833		
		39917	40036		
		40115	40343		
		40554	41213		
		41785	41986		
		42082	42172		
		42341	42398		
		42399	42993		
		43236	43262		
Gene_8.1	Hypothetical protein	43930	44674	-	Supported by EST or cDNA
		44285	44674		
		43930	44284		
Gene_9.1	Kiwellin	46113	47035	+	Supported by EST or cDNA
		46113	46134		
		46135	46776		
		46777	47035		
Gene_10.1	Kiwellin	55484	56128	-	None
		55484	56128		

Gene_11.1	Plant basic secretory protein (BSP) family protein	61885	62541	-	None
		61885	62541		
Gene_12.1	Plant basic secretory protein (BSP) family protein	64485	66020	+	Supported by EST or cDNA
		64485	64504		
		64505	64852		
		65478	65819		
		65820	66020		
Gene_13.1	Nucleic acid binding protein	102509	104587	+	Supported by EST or cDNA
		102509	102590		
		102591	102626		
		102813	102866		
		103748	103867		
		104264	104395		
		104396	104587		
Gene_14.1	Hypothetical protein	104768	114290	-	Supported by EST or cDNA
		114084	114290		
		113560	114083		
		112956	113015		
		111727	111850		
		111540	111608		
		111358	111456		
		110608	110674		
		110338	110402		
		110002	110096		
		109602	109726		
		109417	109517		
		108051	108264		
		107092	107246		
		106862	106984		
		105770	106756		
		105235	105486		
		104768	105234		
Gene_14.2	Hypothetical protein	104768	111538	-	Supported by EST or cDNA
		111484	111538		
		111358	111483		
		110608	110674		
		110338	110402		
		110002	110096		
		109602	109726		
		109417	109517		
		108051	108264		
		107092	107246		
		106862	106984		
		105770	106756		
		105235	105486		
		104768	105234		
Gene_15.1	Glycosyltransferase family 61 protein	118207	120844	-	Supported by EST or cDNA
		120459	120844		
		119924	120335		
		118592	119923		
		118207	118591		
Gene_15.2	Glycosyltransferase family 61 protein	118207	120844	-	Supported by EST or cDNA
		120589	120844		
		120459	120588		
		120210	120335		
		118592	119850		
		118207	118591		
Gene_16.1	Hypothetical protein	122799	128936	-	Supported by EST or cDNA
		128617	128936		
		128150	128616		
		127768	127827		

		127519	127642		
		127341	127385		
		127160	127258		
		125375	125441		
		125087	125151		
		124750	124823		
		124505	124629		
		124391	124410		
		124320	124390		
		122799	123714		
Gene_16.2	Hypothetical protein	122799	128936	-	Supported by EST or cDNA
		128617	128936		
		128150	128616		
		127768	127827		
		127519	127642		
		127341	127385		
		127160	127258		
		125375	125441		
		125087	125151		
		124750	124823		
		124505	124629		
		124320	124420		
		123613	123714		
		122799	123612		
Gene_16.3	Hypothetical protein	122799	128936	-	Supported by EST or cDNA
		128617	128936		
		128150	128616		
		127768	127827		
		127519	127642		
		127341	127385		
		127160	127258		
		125375	125441		
		125087	125151		
		124750	124823		
		124505	124629		
		124353	124420		
		123613	123714		
		122799	123612		
Gene_17.1	Pollen receptor-like kinase 3	132355	135640	+	Supported by EST or cDNA
		132355	132739		
		132740	133688		
		133768	134205		
		134644	135293		
		135294	135640		
Gene_17.2	Pollen receptor-like kinase 3	132355	135640	+	Supported by EST or cDNA
		132355	132739		
		132740	133688		
		133774	134205		
		134644	135293		
		135294	135640		
Gene_18.1	Tonoplast intrinsic protein	136364	137773	+	Supported by EST or cDNA
		136364	136521		
		136522	136672		
		136775	137022		
		137108	137503		
		137504	137773		
Gene_19.1	Hypothetical protein	138432	141476	-	Supported by EST or cDNA
		141375	141476		
		141003	141374		
		140235	140354		
		140098	140151		

		139572	139735		
		139006	139182		
		138714	138924		
		138432	138713		
Gene_20.1	Glycosyl hydrolase, family 13, all-beta	143008	146857	+	Supported by EST or cDNA
		143008	143065		
		143066	143214		
		143421	143482		
		143811	143867		
		144201	144235		
		144369	144472		
		144671	144746		
		144902	144960		
		145066	145131		
		145311	145394		
		145497	145561		
		145673	145755		
		146101	146191		
		146278	146588		
		146589	146857		
Gene_20.2	Glycosyl hydrolase, family 13, all-beta	143008	146857	+	Supported by EST or cDNA
		143008	143186		
		143187	143214		
		143421	143482		
		143811	143867		
		144369	144472		
		144671	144746		
		144902	144960		
		145066	145131		
		145311	145394		
		145497	145561		
		145673	145755		
		146101	146191		
		146278	146588		
		146589	146857		
Gene_20.3	Hypothetical protein	143008	145859	+	Supported by EST or cDNA
		143008	143065		
		143066	143214		
		143421	143482		
		143811	143867		
		144201	144235		
		144369	144472		
		144671	144746		
		144902	144960		
		145066	145131		
		145311	145394		
		145497	145561		
		145673	145785		
		145786	145859		
Gene_21.1	Hypothetical protein	154450	154971	-	None
		154450	154971		
Gene_22.1	Glycosyl hydrolase, family 13, all-beta	166402	174438	+	Supported by EST or cDNA
		166402	166455		
		166456	166655		
		168690	168748		
		171323	171379		
		171541	171575		
		171913	172016		
		172166	172241		
		172341	172399		
		172479	172544		

		172635	172718		
		172800	172864		
		172955	173037		
		173129	173219		
		173304	173353		
		173463	173603		
		173692	173775		
		173776	173797		
		174116	174438		
Gene_22.2	Glycosyl hydrolase, family 13, all-beta	166402	174070	+	Supported by EST or cDNA
		166402	166455		
		166456	166655		
		168690	168748		
		171323	171379		
		171541	171575		
		171913	172016		
		172166	172241		
		172341	172399		
		172479	172544		
		172635	172718		
		172800	172864		
		172955	173037		
		173129	173219		
		173304	173353		
		173463	173603		
		173692	173775		
		173776	174070		
Gene_22.3	Glycosyl hydrolase, family 13, all-beta	170872	174070	+	Supported by EST or cDNA
		170872	171004		
		171005	171035		
		171323	171379		
		171541	171575		
		171913	172016		
		172166	172241		
		172341	172399		
		172479	172544		
		172635	172718		
		172800	172864		
		172955	173037		
		173129	173219		
		173304	173353		
		173463	173603		
		173692	173775		
		173776	174070		
Gene_23.1	Small nuclear RNA activating complex (SNAPc) subunit SNAP43 protein	196644	199359	+	Supported by EST or cDNA
		196644	196939		
		197411	197431		
		197432	197488		
		197568	197694		
		197786	197899		
		198387	198585		
		198703	198748		
		198923	198998		
		199127	199359		
Gene_23.2	Small nuclear RNA activating complex (SNAPc) subunit SNAP43 protein	196885	199650	+	Supported by EST or cDNA
		196885	196939		
		197089	197204		
		197288	197431		
		197432	197488		
		197568	197694		
		197786	197899		

		198387	198585		
		198703	198748		
		198923	198998		
		199127	199359		
		199360	199650		
Gene_24.1	Hypothetical protein	199995	202784	+	Supported by EST or cDNA
		199995	200143		
		200144	200803		
		200900	200947		
		201069	201191		
		201609	201758		
		202249	202420		
		202512	202572		
		202676	202784		
Gene_25.1	Plant/protein (DUF789)	203547	207463	-	Supported by EST or cDNA
		207311	207463		
		206465	206506		
		206289	206464		
		205989	206190		
		205826	205911		
		205440	205531		
		203898	204139		
		203547	203897		
Gene_25.2	Plant/protein (DUF789)	203547	207453	-	Supported by EST or cDNA
		207187	207453		
		207070	207186		
		206289	206506		
		205989	206190		
		205826	205911		
		205440	205531		
		203898	204139		
		203547	203897		
Gene_26.1	N-acetyl-gamma-glutamyl-phosphate reductase, C-terminal part	222730	226504	+	None
		222730	222780		
		223558	223695		
		223907	224014		
		224244	224330		
		224464	224514		
		224611	224682		
		224876	224995		
		225248	225299		
		225421	225501		
		225713	225792		
		225918	226007		
		226172	226300		
		226397	226504		
Gene_27.1	ABC transporter G family member 5	233733	240052	+	Supported by EST or cDNA
		233733	234027		
		234028	234474		
		235848	236137		
		236530	236701		
		236797	236919		
		237021	237149		
		237250	237444		
		237573	237659		
		237752	238024		
		238236	238343		
		238957	239304		
		239305	240052		
Gene_28.1	Hypothetical protein	243547	247017	+	Supported by EST or cDNA
		243547	243555		

		243556	243918		
		244812	244870		
		245678	246764		
		246765	247017		
Gene_29.1	Katanin p80 WD40 repeat-containing subunit B1 homolog	247199	254539	-	Supported by EST or cDNA
		254315	254539		
		254287	254314		
		252221	252351		
		251950	252067		
		251595	251821		
		251345	251447		
		251065	251135		
		250399	250422		
		250191	250265		
		249957	250100		
		249763	249886		
		248708	249646		
		248570	248631		
		248345	248470		
		248189	248263		
		247993	248108		
		247807	247923		
		247630	247698		
		247444	247522		
		247199	247443		
Gene_30.1	KH domain containing protein, expressed	262075	268138	+	Supported by EST or cDNA
		262075	262319		
		264240	264252		
		264253	264876		
		265293	265715		
		265808	266053		
		266163	266903		
		266904	267322		
		267406	267499		
		267598	268138		
Gene_30.2	KH domain containing protein, expressed	262075	268138	+	Supported by EST or cDNA
		262075	262319		
		264240	264252		
		264253	264876		
		265293	265715		
		265808	266053		
		266163	266828		
		267267	267322		
		267406	267499		
		267598	267777		
		267778	268138		
Gene_31.1	Hypothetical protein	283788	284477	+	Supported by EST or cDNA
		283788	283937		
		283938	284477		
Gene_32.1	Hypothetical protein	285748	299545	-	None
		298775	299545		
		294304	294387		
		285748	286470		
Gene_33.1	Hypothetical protein	305142	308859	-	None
		308188	308859		
		305142	305717		
Gene_34.1	Hypothetical protein	310689	310952	-	None
		310689	310952		
Gene_35.1	Chlororespiratory reduction 21	312666	315461	-	None
		312666	315461		
Gene_36.1	Hypothetical protein	317249	321208	-	Supported by EST or cDNA

		320537	321208		
		317491	318066		
		317249	317490		
Gene_37.1	Hypothetical protein	323038	323301	-	None
		323038	323301		
Gene_38.1	Chlororespiratory reduction 21	326700	329495	-	Supported by EST or cDNA
		326700	329495		
Gene_39.1	Hypothetical protein	331497	331997	+	None
		331497	331997		
Gene_40.1	Vacuolar protein-sorting protein bro1	333268	335229	-	None
		334558	335229		
		333268	333978		
Gene_41.1	Endosomal targeting BRO1-like domain-containing protein	342678	347911	-	Supported by EST or cDNA
		347795	347911		
		347123	347794		
		345593	346543		
		345337	345426		
		345178	345246		
		344261	344401		
		343935	343983		
		343379	343482		
		342678	343253		
Gene_42.1	Nucleolar complex protein 2-like protein	356871	362049	-	Supported by EST or cDNA
		361987	362049		
		361965	361986		
		361752	361876		
		360229	360300		
		359624	360133		
		359202	359516		
		359052	359108		
		358550	358876		
		358264	358470		
		358100	358186		
		357920	358014		
		357567	357701		
		357160	357481		
		356871	357159		
Gene_43.1	DNA-directed RNA polymerase, beta subunit	362770	374254	-	Supported by EST or cDNA
		374105	374254		
		372502	372578		
		372173	372236		
		371995	372065		
		371794	371871		
		371658	371703		
		371063	371243		
		370924	370976		
		370765	370838		
		370555	370672		
		369703	369787		
		368375	368487		
		367361	367488		
		367206	367285		
		366917	367116		
		366621	366785		
		366381	366545		
		366184	366258		
		365506	365609		
		365348	365423		
		364602	364724		
		364349	364492		
		363822	364034		

		363417	363650		
		362770	363213		
Gene_44.1	Aerobic coproporphyrinogen-III oxidase (DUF1218)	376061	379266	+	Supported by EST or cDNA
		376061	376164		
		376165	376257		
		376378	376583		
		378658	378910		
		378911	379266		
Gene_45.1	Dof domain, zinc finger family protein, expressed	379695	381191	-	Supported by EST or cDNA
		380809	381191		
		380707	380808		
		379987	380628		
		379695	379986		
Gene_46.1	Hypothetical protein	388850	389707	-	None
		388850	389707		
Gene_47.1	Hypothetical protein	390174	402348	-	Supported by EST or cDNA
		402238	402348		
		401944	402152		
		401544	401838		
		401369	401467		
		400923	401296		
		392275	392326		
		391267	391357		
		390174	390364		
Gene_48.1	Short-chain dehydrogenase TIC 32, chloroplastic	412588	417637	+	Supported by EST or cDNA
		412588	412667		
		412668	412730		
		412871	412957		
		414179	414710		
		416639	417165		
		417166	417637		
Gene_49.1	Hypothetical protein	420465	428010	+	None
		420465	420946		
		422272	422371		
		422725	423058		
		423329	423441		
		424323	424451		
		426548	426837		
		426984	427128		
		427762	427859		
		427968	428010		
Gene_50.1	WAPL (Wings apart-like protein regulation of heterochromatin) protein	430950	434247	+	Supported by EST or cDNA
		430950	431144		
		431145	431220		
		431330	431515		
		432122	432528		
		432876	432977		
		433084	433311		
		433312	433389		
		434049	434247		
Gene_51.1	Hypothetical protein	447478	447759	-	None
		447478	447759		
Gene_52.1	Pentatricopeptide repeat protein	449577	457039	+	Supported by EST or cDNA
		449577	449682		
		449683	451503		
		456995	457039		
Gene_53.1	Pentatricopeptide repeat protein	460462	462716	+	Supported by EST or cDNA
		460462	462513		
		462514	462716		
Gene_54.1	Pentatricopeptide repeat protein	464579	468164	+	Supported by EST or cDNA
		464579	464832		

		464833	466580		
		467990	468164		
Gene_55.1	Pentatricopeptide repeat protein	472831	475164	+	None
		472831	475164		
Gene_56.1	Probable receptor-like serine/threonine-protein kinase At4g34500	489106	493840	+	Supported by EST or cDNA
		489106	489606		
		490034	490050		
		490051	490718		
		490811	490915		
		492090	492212		
		492329	492499		
		492673	492802		
		493055	493264		
		493354	493476		
		493477	493840		
Gene_57.1	Protein COBRA	494625	497177	+	Supported by EST or cDNA
		494625	494767		
		494965	495044		
		495608	495938		
		495939	496165		
		496269	496543		
		496726	496949		
		496950	497177		
Gene_58.1	Prolyl 4-hydroxylase 1	499446	503382	+	Supported by EST or cDNA
		499446	499570		
		499571	499857		
		499985	500075		
		500769	500866		
		500951	501048		
		502343	502472		
		502561	502748		
		502847	502920		
		502921	502944		
		503091	503382		
Gene_59.1	LanC-like protein GCR2	504862	507165	+	Supported by EST or cDNA
		504862	505000		
		505001	505138		
		505279	505457		
		505579	505696		
		505819	505935		
		506039	506353		
		506569	506907		
		506908	507165		
Gene_59.2	LanC-like protein GCR2	504869	507165	+	Supported by EST or cDNA
		504869	505000		
		505001	505138		
		505279	505457		
		505585	505696		
		505819	505935		
		506039	506353		
		506569	506907		
		506908	507165		
Gene_60.1	Epoxide hydrolase	507496	509609	-	Supported by EST or cDNA
		509286	509609		
		509213	509285		
		508896	509113		
		508464	508625		
		508082	508321		
		507496	508081		
Gene_60.2	Epoxide hydrolase	507496	509609	-	Supported by EST or cDNA
		509286	509609		

		509213	509285		
		508896	509113		
		508464	508625		
		508086	508321		
		507710	507956		
		507496	507709		
Gene_61.1	Epoxide hydrolase	512849	514926	+	Supported by EST or cDNA
		512849	512936		
		513045	513424		
		513511	513746		
		514384	514642		
		514643	514926		
Gene_62.1	Epoxide hydrolase	515904	518142	+	Supported by EST or cDNA
		515904	515987		
		515988	516072		
		516160	516377		
		516974	517135		
		517218	517453		
		517541	517796		
		517797	518142		
Gene_63.1	Epoxide hydrolase	519249	521204	+	Supported by EST or cDNA
		519249	519330		
		519331	519418		
		519512	519729		
		520245	520406		
		520484	520719		
		520807	521062		
		521063	521204		
Gene_63.2	Epoxide hydrolase	519249	521204	+	Supported by EST or cDNA
		519249	519576		
		519577	519729		
		520245	520406		
		520484	520719		
		520807	521062		
		521063	521204		
Gene_64.1	Epoxide hydrolase	522853	524793	+	Supported by EST or cDNA
		522853	522964		
		522965	523064		
		523216	523433		
		523852	524013		
		524092	524327		
		524416	524671		
		524672	524793		
Gene_65.1	Protein STABILIZED1	526221	530814	-	Supported by EST or cDNA
		530759	530814		
		527639	530758		
		527612	527638		
		526221	526693		
Gene_66.1	Mediator of RNA polymerase II transcription subunit 16	535879	541963	+	Supported by EST or cDNA
		535879	535886		
		536291	536298		
		536299	536909		
		536994	537073		
		537699	537781		
		538321	538557		
		539007	539184		
		539342	539856		
		540268	540444		
		540913	541296		
		541413	541559		
		541560	541963		

Gene_66.2	Mediator of RNA polymerase II transcription subunit 16	535879	541963	+	Supported by EST or cDNA
		535879	535886		
		536291	536298		
		536299	536909		
		536994	537073		
		537699	537781		
		538321	538557		
		539007	539184		
		539342	539856		
		540268	540444		
		540913	541296		
		541413	541559		
		541560	541588		
		541688	541963		
Gene_67.1	UPF0533 C5orf44-like protein	544597	550101	+	Supported by EST or cDNA
		544597	544670		
		544671	545059		
		545152	545236		
		545906	546036		
		546137	546239		
		546384	546398		
		546854	546999		
		547122	547317		
		547440	547514		
		548243	548359		
		548886	548930		
		549450	549548		
		549786	549806		
		549807	550101		
Gene_68.1	Hypothetical protein	550617	552613	-	None
		552503	552613		
		552213	552368		
		551190	551303		
		550617	550901		
Gene_69.1	CBS domain-containing protein CBSX5	568670	571126	+	Supported by EST or cDNA
		568670	568857		
		568858	569189		
		570022	570847		
		570848	571126		
Gene_70.1	Pentatricopeptide repeat protein	571908	574704	+	Supported by EST or cDNA
		571908	572048		
		572126	572161		
		572162	573682		
		573683	574704		
Gene_71.1	Hypothetical protein	578164	580900	+	None
		578164	578207		
		578330	578477		
		578568	578614		
		579226	579280		
		579375	579413		
		579527	579698		
		580154	580211		
		580389	580445		
		580648	580900		
Gene_72.1	Hypothetical protein	585438	585820	-	None
		585616	585820		
		585438	585472		
Gene_73.1	Transparent testa 2 family isoform 2	586202	588140	-	None
		588038	588140		
		587300	587429		
		586202	586853		

Gene_74.1	Ubiquitin-protein ligase/ zinc ion binding protein	593090	596037	+	Supported by EST or cDNA
		593090	593314		
		593315	593379		
		593869	594019		
		594107	594173		
		594281	594515		
		595466	595676		
		595677	596037		
Gene_74.2	Ubiquitin-protein ligase/ zinc ion binding protein	593090	596037	+	Supported by EST or cDNA
		593090	593379		
		593869	594024		
		594025	594039		
		594107	594173		
		594281	594515		
		595466	595676		
		595677	596037		
Gene_75.1	Acetyltransferase	596900	597636	-	Supported by EST or cDNA
		597591	597636		
		597287	597590		
		596977	597197		
		596900	596976		
Gene_76.1	30S ribosomal protein S18	598690	602062	+	Supported by EST or cDNA
		598690	598803		
		598804	598930		
		599494	599789		
		600158	600253		
		600885	600944		
		601489	601734		
		601735	602062		
Gene_77.1	Hypothetical protein	607057	610096	+	Supported by EST or cDNA
		607057	607367		
		607550	607679		
		607680	608825		
		609155	609376		
		609491	609610		
		609691	609744		
		609745	610096		
Gene_77.2	Hypothetical protein	607057	610057	+	Supported by EST or cDNA
		607057	607367		
		607550	607679		
		607680	608825		
		609155	609376		
		609491	609610		
		609700	609744		
		609745	610057		
Gene_77.3	Hypothetical protein	607057	610057	+	Supported by EST or cDNA
		607057	607367		
		607550	607679		
		607680	608825		
		609155	609376		
		609491	609622		
		609623	610057		
Gene_77.4	Hypothetical protein	607057	610057	+	Supported by EST or cDNA
		607057	607367		
		607550	607679		
		607680	608825		
		609155	609451		
		609452	609610		
		609700	610057		
Gene_78.1	Monothiol glutaredoxin-S17	611259	615023	+	Supported by EST or cDNA
		611259	611398		

		611399	611566		
		612722	612796		
		613458	614690		
		614691	615023		
Gene_79.1	Pentatricopeptide repeat-containing protein mitochondrial	624228	625894	+	Supported by EST or cDNA
		624228	624553		
		624662	624667		
		624668	625693		
		625694	625894		
Gene_80.1	Pentatricopeptide repeat-containing protein At1g02150	632378	637802	+	Supported by EST or cDNA
		632378	632482		
		632483	632791		
		632876	634108		
		637498	637802		
Gene_80.2	Pentatricopeptide repeat-containing protein At1g02150	632378	634454	+	Supported by EST or cDNA
		632378	632482		
		632483	632791		
		632876	634108		
		634109	634454		
Gene_81.1	Calcium uniporter protein 5 mitochondrial	650110	653853	+	Supported by EST or cDNA
		650110	650237		
		650238	650732		
		652882	653400		
		653401	653853		

Table S5 Seed setting rates and anther phenotypes of PPR-transgenic TAA lines

Line	Plant No.	Filled spiklets / Total spikelets			Ave. (%)	(±) S.D.	Anther phenotype*
		Panicle 1	Panicle 2	Panicle 3			
PPR796	1	61/66	59/69	60/63	91.1	4.1	D
	2	31/75	47/91	20/50	44.3	5.2	D
	3	0/54	0/66	0/59	0.0	0.0	I
	4	28/32	45/49	58/66	89.1	2.0	D
	5	40/51	58/60	37/38	90.8	8.8	D
	6	0/54	3/20	3/25	9.0	6.5	I
	7	54/56	57/60	77/79	96.3	1.0	D
	8	49/50	35/40	57/59	94.0	4.7	D
	9	2/21	1/39	2/29	6.3	2.9	I
	10	39/52	61/78	41/61	73.5	4.6	D
	11	12/47	5/51	3/49	13.8	8.4	D
	12	10/38	10/53	4/25	20.4	4.3	D
	13	1/48	3/64	2/67	3.3	1.1	I
	14	0/39	0/69	0/59	0.0	0.0	D
	15	17/49	18/28	39/61	54.3	13.9	D
	16	35/45	41/48	66/70	85.8	6.7	D
	17	12/22	9/36	6/39	31.6	16.7	D
	18	0/35	4/51	1/47	3.3	3.3	I
	19	68/73	68/83	47/54	87.4	4.6	D
	20	38/50	37/73	46/70	64.1	10.4	D
	21	51/61	48/52	48/57	86.7	4.0	D
	22	0/20	0/25	0/32	0.0	0.0	I
	23	7/27	6/30	10/33	25.4	4.2	D
	24	55/58	60/64	32/38	90.9	4.8	D
	25	2/40	8/70	0/56	5.5	4.7	D
	26	7/72	4/84	5/51	8.1	2.4	I
	27	18/30	69/87	65/74	75.7	11.6	D
PPR782	1	2/84	1/92	0/99	1.2	1.0	I
	2	0/30	2/49	1/61	1.9	1.7	I
	3	0/62	0/51	0/66	0.0	0.0	I
	4	0/59	0/55	0/50	0.0	0.0	I
	5	0/70	3/61	0/48	1.6	2.3	I
	6	0/80	0/75	0/69	0.0	0.0	I
	7	0/35	0/43	0/65	0.0	0.0	I
	8	0/60	1/55	8/70	4.4	5.0	I
PPR762	1	0/98	4/66	0/51	2.0	2.9	I
	2	1/141	0/59	0/73	0.2	0.3	I
	3	1/120	2/45	1/46	2.5	1.5	I
	4	0/35	0/15	0/30	0.0	0.0	I
	5	2/75	5/80	1/62	3.5	2.0	I
	6	4/85	0/157	5/65	4.1	3.2	I
	7	6/60	1/99	13/81	9.0	6.2	I
	8	1/98	1/123	0/101	0.6	0.4	I
PPR683	1	0/20	0/94	4/112	1.2	1.7	I
	2	0/119	0/50	0/55	0.0	0.0	I
	3	7/80	3/69	7/95	6.8	1.8	I
	4	0/90	0/53	0/62	0.0	0.0	I
	5	0/31	0/28	0/58	0.0	0.0	I
	6	0/23	1/38	0/36	0.9	1.2	I
	7	0/59	0/40	0/38	0.0	0.0	I
PPR777	1	0/30	0/48	0/43	0.0	0.0	I
	2	0/97	0/111	0/108	0.0	0.0	I
	3	0/50	0/51	0/45	0.0	0.0	I
	4	0/20	0/53	0/54	0.0	0.0	I
	5	0/40	0/40	0/31	0.0	0.0	I
	6	0/54	0/51	0/69	0.0	0.0	I
	7	0/56	2/50	0/50	1.3	1.9	I
PPR794	1	3/140	0/63	0/78	0.7	1.0	I
	2	0/19	1/39	0/80	0.9	1.2	I
	3	0/29	0/25	2/67	1.0	1.4	I
	4	1/30	0/28	0/37	1.1	1.6	I
	5	1/35	1/53	0/79	1.6	1.2	I
	6	0/35	0/54	2/73	0.9	1.3	I
	7	0/53	0/66	0/55	0.0	0.0	I
	8	0/61	0/54	0/32	0.0	0.0	I
	9	0/129	0/113	0/98	0.0	0.0	I
	10	0/110	0/88	0/75	0.0	0.0	I
	11	0/84	0/55	0/49	0.0	0.0	I
	12	0/69	0/99	0/105	0.0	0.0	I
T65	1	73/75	88/95	103/105	96.0	2.4	D
	2	102/109	85/86	110/113	96.6	2.2	D
	3	59/60	78/83	80/88	94.4	3.0	D
TAA	1	0/66	1/82	1/77	0.8	0.6	I
	2	0/60	0/67	0/55	0.0	0.0	I
	3	3/56	0/79	9/82	5.4	4.5	I
TAR	1	82/84	85/91	91/100	94.0	2.7	D
	2	75/78	74/80	96/107	92.8	2.6	D
	3	67/74	79/86	92/107	89.5	2.5	D

*Anther phenotype was judged based on the predominant appearance of dehiscent (D) and indehiscent (I) anthers in Figs.S4-S6.

Table S6 Predicted sequences of the PPR796-binding site (BS) on *orf312* transcript

Start*	End*	p-value	q-value	Matched Sequence
+824	+841	6.72E-05	0.138	GGUUGCAUCAAAUGCUUG
+711	+728	2.30E-04	0.236	GGUUGACAUAAUCCGGAA
+86	+103	2.07E-03	0.971	UACUGUUUCUUAUUCUUA
+944	+961	2.79E-03	0.971	GGCUGAGAGAAGGGUGAA
+699	+716	2.84E-03	0.971	UUUUGAGGUGAAGGUUGA

* Position was counted from the initiation codon of *orf312* .

Table S7 Number of clones in RACE analysis

Line	Position*	Number
TAA	+1082	1
	+1086	1
	+1088	3
	+1089	5
	+1090	2
	+1095	2
	+1098	1
PPR796- transgenic line #2	+858	2
	+865	1
	+872	1
	+920	5
	+922	1
	+964	2
	+1055	1
	+1088	3
	+1089	1
	+1090	1
	+1098	1
TAR	+848	1
	+858	1
	+901	1
	+904	1
	+919	2
	+920	6
	+921	1
	+922	2
	+972	1

* Position was counted from the initiation codon of *orf312* .
